# Morpho-Physicochemical, Bioactive, and Antioxidant Profiling of Peruvian *Coffea arabica* L. Germplasm Reveals Promising Accessions for Agronomic and Nutraceutical Breeding

**DOI:** 10.3390/plants15010013

**Published:** 2025-12-19

**Authors:** César Cueva-Carhuatanta, Ester Choque-Incaluque, Ronald Pio Carrera-Rojo, Jazmín Maravi Loyola, Marián Hermoza-Gutiérrez, Hector Cántaro-Segura, Elizabeth Fernandez-Huaytalla, Dina L. Gutiérrez-Reynoso, Fredy Quispe-Jacobo, Karina Ccapa-Ramirez

**Affiliations:** 1 Centro Experimental La Molina, Dirección de Recursos Genéticos y Biotecnología, Instituto Nacional de Innovación Agraria (INIA), Av. La Molina 1981, Lima 15024, Peru; cesar.cueva01c@gmail.com (C.C.-C.); esmaunfv@gmail.com (E.C.-I.); dr.marian.hermoza@gmail.com (M.H.-G.); hcantarosegura@gmail.com (H.C.-S.); efernandezh@inia.gob.pe (E.F.-H.); dgutierrez@inia.gob.pe (D.L.G.-R.); 2 Estación Experimental Agraria Pichanaki, Dirección de Recursos Genéticos y Biotecnología, Instituto Nacional de Innovación Agraria (INIA), Carretera Marginal Km. 74, Pichanaqui 12866, Peru; rcarrera@inia.gob.pe (R.P.C.-R.); jmaravi@inia.gob.pe (J.M.L.); 3 Laboratorio de Investigación Nutricional de Los Recursos Genéticos, Dirección de Recursos Genéticos y Biotecnología, Instituto Nacional de Innovación Agraria (INIA), Av. La Molina 1981, Lima 15024, Peru; fredyenrique@gmail.com

**Keywords:** *Coffea arabica*, coffee germplasm, bioactive, antioxidant activity, phenolic compounds, chlorogenic acid

## Abstract

Coffee quality arises from the interaction among genotype, environment, and postharvest management, yet few large-scale studies jointly integrate agronomic, phytochemical, and processing traits. We characterized 150 *Coffea arabica* L. accessions from six Peruvian regions, evaluated in the INIA coffee germplasm collection, quantifying agro-morphological traits, colorimetric parameters in cherries and beans, fermentation indicators, bioactive compounds, and antioxidant activity. Correlation analyses showed that total phenolics (TPCs) and total flavonoids (TFCs) were strongly associated with antioxidant activity, whereas caffeine content (CAF) varied, largely independently. Several chromatic parameters in parchment and green coffee (a*, b*, C*) showed positive correlations with phenolic content and antioxidant activity (ABTS, DPPH, FRAP), while final fermentation pH (FPH) was negatively associated with these compounds, supporting both color metrics and pH as operational indicators of chemical quality. Principal component analysis disentangled a morphometric gradient from a functional (phenolic–antioxidant) gradient, indicating that bean size and antioxidant potential can be improved in a semi-independent manner. Hierarchical clustering identified complementary ideotypes, and a multi-trait selection index highlighted promising accessions—PER1002197 (Cajamarca), PER1002222 (Cajamarca), PER1002288 (Pasco), and PER1002184 (Cajamarca)—that combine high phenolic/antioxidant levels, favorable chlorogenic acid (CGA)/trigonelline (TGN) profiles, contrasting (high/low) caffeine, and competitive yield (YPP)/bean size. These accessions represent promising candidates for breeding climate-smart and nutraceutical-oriented coffee.

## 1. Introduction

Coffee (*Coffea* spp.), belonging to the family Rubiaceae, ranks among the most economically valuable tropical crops, sustaining the livelihoods of over 25 million farmers worldwide and contributing substantially to the economies of developing nations [1,2]. Among cultivated species, *Coffea arabica* L. is prized for its superior organoleptic quality and accounts for approximately 60% of global coffee production [3]. Native to the East African highlands, *C. arabica* has been introduced and naturalized across a broad spectrum of environments, including Peru’s heterogeneous agroecological landscapes [4]. In this Andean country, coffee is grown across diverse climates and elevations, from lowland Amazonian valleys to warm inter-Andean basins, forming a mosaic of genetic and phenotypic diversity that offers opportunities for genetic improvement and pathways to improved livelihoods for coffee growers [5,6].

Green coffee contains multiple bioactives, most notably chlorogenic acids, trigonelline, and caffeine, that shape antioxidant activity and the development of beverage flavor upon roasting [7,8,9]. These phytochemicals are modulated by genotype, environmental conditions, and agronomic management [10,11,12], as well as by postharvest processing [13], in which fermentation parameters are critical, and ripeness, proxied by fruit color, plays a key role [14,15,16,17]. In this context, recent breeding efforts increasingly prioritize secondary metabolites and antioxidant activity as selection criteria for elite materials, aligning with rising consumer demand for functional and health-promoting foods [18,19].

Studies on national coffee germplasm collections, whether conserved in situ or ex situ, remain scarce. Preliminary evaluations in Brazil and Ethiopia reported substantial variability in bioactive compounds and cup quality, linking these traits to genetic improvement pathways [20,21]. In Peru, however, the assessments by Paredes-Espinoza et al. [22] and Alvarado et al. [23] focused primarily on agronomic characterization and did not integrate morphological descriptors with physicochemical parameters, secondary metabolites, antioxidant activity, or cup quality. This lack of integration constrains the deployment of modern tools such as marker-assisted selection and genomic prediction, which constitute the next steps toward accelerating coffee breeding [19].

Addressing this gap requires integrated phenotyping to identify promising ideotypes for conservation and genetic improvement [24,25,26,27], an especially compelling opportunity in Peru, given its pronounced altitudinal zonation and heterogeneous microclimates. This approach leverages environmental gradients as natural experiments to elucidate how the interaction between environmental factors and genetic background modulates bean development, color expression, fermentation behavior, and phytochemical composition [28,29,30,31].

We hypothesize that the *Coffea arabica* L. accessions from the Peruvian germplasm collection exhibit sufficient morpho-physicochemical and phytochemical variation to establish associations among productive traits, postharvest quality indicators, bioactive composition, and antioxidant activity. Such variability would allow the identification of functional ideotypes integrating productivity and potential nutraceutical quality, providing a basis for breeding programs aimed at developing varieties with high agronomic and functional value.

In this study, we characterized 150 *Coffea arabica* L. accessions from the National Coffee Germplasm Collection of the Instituto Nacional de Innovación Agraria (INIA), originating from six coffee-growing regions of Peru. We evaluated agro-morphological traits (yield per plant; dimensions and weight of parchment and green beans); fruit, parchment, and green-bean color in the CIELAB space; fermentation indicators (soluble solids content, IB/FB and pH, IPH/FPH); bioactive compounds (total phenolics, total flavonoids, chlorogenic acids, trigonelline, and caffeine); and antioxidant activity. Multivariate analyses (correlations, PCA, and Ward’s hierarchical clustering) were used to resolve trait modules and ideotypes, and a multi-trait selection index (1–5 scale per trait) was implemented to rank accessions by combined agronomic and nutraceutical merit.

The aim was to identify promising materials within the INIA national collection for breeding and for the development of functional, nutraceutical-oriented coffees through comprehensive phenotypic characterization of agro-morphological traits, postharvest physicochemical attributes, bioactive profiles, and antioxidant activity. Integrating these dimensions at population scale establishes a phenotypic foundation for conserving and harnessing Peru’s coffee diversity to develop high-quality cultivars with functional value for human health.

## 2. Results

The characterization of the 150 coffee accessions revealed substantial variability across the evaluated parameters; descriptive statistics are provided in Table 1.

### 2.1. Agro-Morphologic Characterization

Fruit yield per plant exhibited wide dispersion within the agro-morphological set, ranging from 0.14 to 2.64 kg plant^−1^ (mean 0.90 ± 0.55 kg plant^−1^; CV = 61.6%), with a positively skewed distribution indicative of a minority of highly productive genotypes. Coffee fruit dimensions were variable: length ranged from 12.55 to 20.18 mm (15.53 ± 1.00 mm), width from 11.45 to 15.69 mm (13.66 ± 0.82 mm), and thickness from 10.13 to 14.18 mm (11.83 ± 0.73 mm).

Parchment coffee weight ranged from 0.14 to 0.37 g (0.19 ± 0.03 g). Parchment bean length, width, and thickness varied across 8.77–16.17 mm (11.98 ± 1.02 mm), 6.76–9.38 mm (7.89 ± 0.38 mm), and 4.24–5.83 mm (4.78 ± 0.27 mm), respectively. Green bean weight spanned 0.10–0.28 g (0.16 ± 0.03 g). Green bean dimensions also varied: length 7.93–13.00 mm (9.63 ± 0.77 mm), width 5.92–8.20 mm (6.72 ± 0.33 mm), and thickness 3.12–4.20 mm (3.68 ± 0.20 mm).

Dispersion in parchment and green bean dimensions was low (CV = 4.8–8.5%), whereas bean weight concentrated most of the morphological variance after yield (CV = 16%).

Table 1 reveals a broad variability pattern within the agro-morphological block: (i) fruit yield per plant, bean weight, and fruit color are the most discriminating traits (high CVs, non-normal distributions), offering a practical starting point for selection; and (ii) linear dimensions of fruit and seed exhibit low variability, providing desirable morphological stability for processing and grading. This distributional heterogeneity is consistent with the multivariate separation observed across parameters.

### 2.2. Color Characterization and Physicochemical Parameters

Cherry color exhibited the greatest dispersion among CIELAB measurements. Lightness (L*) averaged 37.50 (range 30.54–60.05; CV = 20.0%), a* averaged 22.16 (−0.97 to 30.81; CV = 33.74%), b* averaged 16.82 (8.38–44.70; CV = 53.49%), chroma (C*) averaged 29.92 (20.61–45.00; CV = 16.3%), and hue angle (*h°*) averaged 35.29 (21.17–91.61; CV = 53.9%). The wide ranges and elevated coefficients of variation—particularly for b* and *h°*—indicate marked phenotypic diversity in cherry pigmentation, spanning hues from red to yellow.

In parchment coffee, color was more uniform. Lightness was high and narrowly distributed (L* = 51.26; 46.23–55.61; CV = 3.34%). The a* coordinate remained low (2.38; 0.55–4.56; CV = 36.5%), whereas b* and C* showed moderate variability (b* = 17.81; 13.09–21.78; CV = 8.26%; C* = 17.99; 13.14–22.17; CV = 8.61%). Hue angle clustered near the yellow region (*h°* = 82.56; 77.06–87.94; CV = 2.78%), evidencing a homogeneous parchment tone after drying.

In green coffee, color values were approximately normally distributed around the mean, except for a*. Lightness averaged 44.60 (39.01–48.88; CV = 3.54%); a* values were near zero (0.94; 0.39–1.59; CV = 25.29%); and b* and C* were moderate (b* = 9.92; 6.72–12.07; CV = 8.55%; C* = 9.97; 6.76–12.15; CV = 8.55%). Hue angle was stable around yellow-green (*h°* = 84.64; 81.20–87.72; CV = 1.53%). Taken together, color variability is greatest in coffee cherries and decreases progressively from parchment to green beans, reflecting increasing product standardization at each postharvest stage.

Total soluble solids in fresh fruit ranged from 15.23 to 24.50 °Brix (mean 19.31 ± 1.96 °Brix). During fermentation, these values declined significantly to 8.53–16.13 °Brix (12.75 ± 1.72 °Brix). Initial pH ranged from 4.44 to 6.28 (5.25 ± 0.39) and decreased to 3.08–4.78 at the end of fermentation (4.03 ± 0.36), consistent with the acidification characteristic of wet processing.

Green coffee moisture averaged 6.89 ± 0.64% (5.22–8.64%; CV = 9.35%), values that ensure product stability and safety.

### 2.3. Bioactive Compounds and Antioxidant Activity

Total phenolics averaged 45.53 mg GAE g^−1^ (range 32.73–57.78 mg GAE g^−1^; CV = 8.94%) with an approximately symmetric distribution. Total flavonoids showed comparable magnitude and dispersion, with a mean of 40.01 mg CE g^−1^ (28.82–50.68 mg CE g^−1^; CV = 9.53%), slight negative skewness, and an approximately normal distribution. Taken together, these metrics indicate moderate dispersion in total phenolic and flavonoid contents across accessions.

Antioxidant activity spanned a broader range depending on the assay. ABTS values averaged 206.00 μmol TE g^−1^ (146.50–259.60 μmol TE g^−1^; CV = 10.3%) and were close to symmetric. By contrast, DPPH exhibited greater dispersion, with a mean of 223.70 μmol TE g^−1^ (114.70–321.10 μmol TE g^−1^; CV = 18.43%), negative skewness, and an approximately normal distribution. FRAP showed the highest variability among the three assays, averaging 383.8 μmol Fe^2+^ g^−1^ (156.30–520.40 μmol Fe^2+^ g^−1^; CV = 19.17%), with negative skewness and a marked departure from normality. These results indicate substantial variability in radical-scavenging and reducing capacities, with ABTS being comparatively homogeneous, whereas FRAP and DPPH display wider extremes.

Regarding biologically active secondary metabolites (quantified by liquid chromatography), chlorogenic acids averaged 42.88 mg g^−1^ (30.50–55.42 mg g^−1^; CV = 12.85%) with near-symmetric distribution; trigonelline averaged 12.08 mg g^−1^ (9.04–15.74 mg g^−1^; CV = 12.52%); and caffeine averaged 11.84 mg g^−1^ (8.57–15.39 mg g^−1^; CV = 9.96%). Thus, chlorogenic acids spanned the widest range among the metabolites, whereas caffeine remained comparatively conserved. Overall, the phytochemical block reveals moderate-to-high chemical diversity among accessions: phenolics and ABTS are relatively homogeneous, while FRAP, DPPH, chlorogenic acids, and trigonelline introduce greater dispersion, providing broad contrast for identifying accessions with enhanced functional profiles.

### 2.4. Correlation Analysis

**Figure 1:**
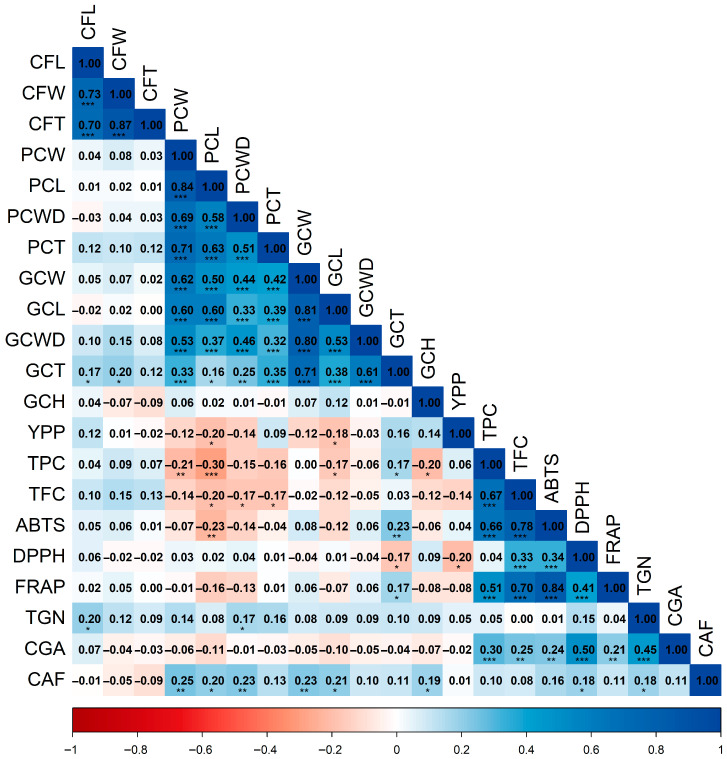
Spearman correlation matrix of pairwise associations among agro-morphological parameters, bioactive compounds, and antioxidant activity. Abbreviations: CFL, fruit length; CFW, fruit width; CFT, fruit thickness; PCW, parchment coffee weight; PCL, parchment coffee length; PCWD, parchment coffee width; PCT, parchment coffee thickness; GCW, green coffee weight; GCL, green coffee length; GCWD, green coffee width; GCT, green coffee thickness; GCH, green coffee moisture; YPP, yield per plant; TPC, total phenolic content; TFC, total flavonoid content; ABTS, DPPH, FRAP, antioxidant activity assays; TGN, trigonelline; CGA, chlorogenic acids; CAF, caffeine. Significance: *p* < 0.05 (*); *p* < 0.01 (**); *p* < 0.001 (***).

The correlation analysis revealed significant relationships among the agro-morphological traits, biochemical compounds, and antioxidant activity evaluated across the 150 coffee accessions. A first Spearman correlation matrix was constructed using 21 variables to associate agro-morphological traits with phytochemicals (Figure 1).

The Spearman heatmap (Figure 1) resolves three coherent association blocks. First, fruit and bean size traits are tightly intercorrelated. Fruit length, width, and thickness (CFL–CFW–CFT) showed strong positive correlations (0.70–0.87). In parchment coffee, weight correlated closely with length and thickness (PCW–PCL, 0.84; PCW–PCT, 0.71), whereas in green coffee, weight was strongly related to length, and the transverse dimensions were mutually associated (GCW–GCL, 0.81; GCWD–GCT, 0.61). Second, a functional (bioactive/antioxidant) module emerges. Total phenolics covaried strongly with total flavonoids (TPC–TFC, 0.67, *p* < 0.001) and aligned positively with antioxidant activity (TPC–ABTS, 0.66; TPC–FRAP, 0.51; *p* < 0.001), but showed only a negligible association with DPPH (TPC–DPPH, 0.04). Flavonoids followed the same pattern (TFC–FRAP, 0.70; TFC–ABTS, 0.78; TFC–DPPH, 0.33; *p* < 0.001). The antioxidant assays were mutually consistent and highly significant (ABTS–FRAP, 0.84; DPPH–FRAP, 0.41; *p* < 0.001). Collectively, these correlations indicate that phenolics are the principal drivers of the measured antioxidant responses—particularly ferric-reducing capacity and ABTS radical scavenging. Third, specific secondary metabolites, notably chlorogenic acids (CGA), associated with antioxidant activity: CGA correlated positively with the antioxidant block (CGA–FRAP, 0.21; CGA–DPPH, 0.50; CGA–ABTS, 0.24; *p* < 0.01) and with phenolics (0.30), flavonoids (0.25), and trigonelline (0.45; all *p* < 0.01). By contrast, caffeine (CAF) displayed only a modest positive relationship with DPPH (0.18; *p* < 0.05), consistent with an otherwise independent accumulation pattern.

Regarding size traits, parchment and green coffee showed significant negative correlations (*p* < 0.05) with phenolics (TPC–PCW, −0.21; TPC–PCL, −0.30; TPC–GCL, −0.17) and flavonoids (TFC–PCL, −0.20; TFC–PCT, −0.17; TFC–PCWD, −0.17), suggesting that, on average, larger beans tend to exhibit slightly lower concentrations of these compounds.

**Figure 2:**
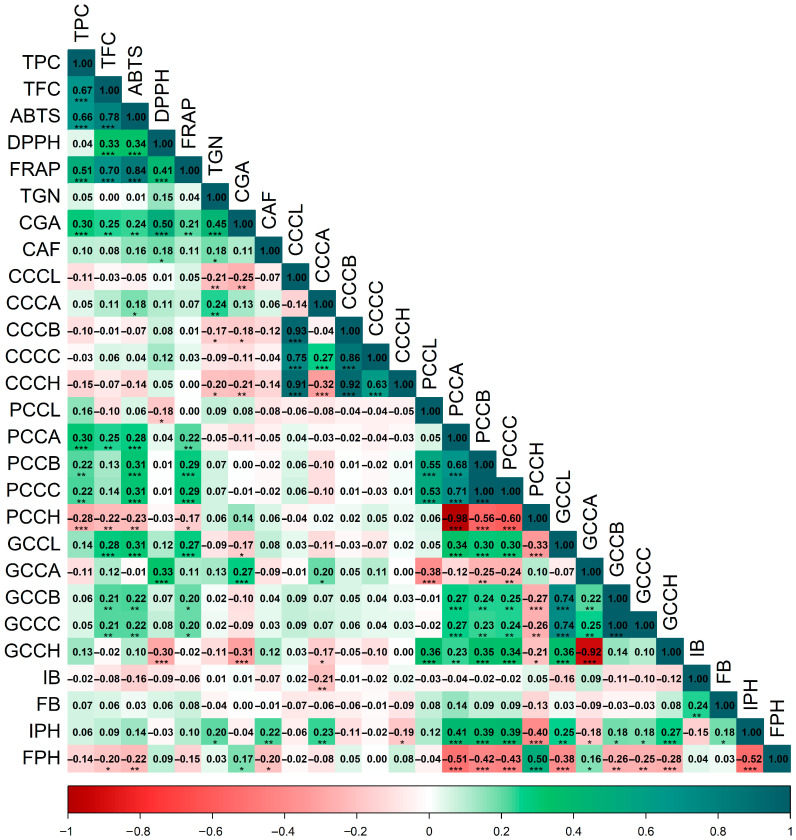
Spearman correlation matrix of pairwise associations among bioactive, color, and fermentation parameters. Abbreviations: CCCL, coffee cherry color L*; CCCA, coffee cherry color a*; CCCB, coffee cherry color b*; CCCC, coffee cherry color C*; CCCH, coffee cherry color *h°*; PCCL, parchment coffee color L*; PCCA, parchment coffee color a*; PCCB, parchment coffee color b*; PCCC, parchment coffee color C*; PCCH, parchment coffee color *h°*; GCCL, green coffee color L*; GCCA, green coffee color a*; GCCB, green coffee color b*; GCCC, green coffee color C*; GCCH, green coffee color *h°*; IB, initial soluble solids (°Brix); FB, final soluble solids (°Brix); IPH, initial pH; FPH, final pH; TPC, total phenolic content; TFC, total flavonoid content; ABTS, DPPH, FRAP, antioxidant activity assays; TGN, trigonelline; CGA, chlorogenic acids; CAF, caffeine. Significance: *p* < 0.05 (*); *p* < 0.01 (**); *p* < 0.001 (***).

A second Spearman correlation matrix (Figure 2) integrating bioactive/antioxidant traits with color (CIELAB) and fermentation variables reveals consistent patterns across cherry, parchment, and green coffee. Color coordinates were strongly interrelated: in cherries, color parameters showed highly significant associations (*p* < 0.001) (CCCA–CCCC = 0.27; CCCA–CCCH = −0.32; CCCB–CCCC = 0.86; CCCC–CCCH = 0.63; CCCB–CCCH = 0.92); in parchment coffee, a*, b*, and C* (PCCA/PCCB/PCCC) were highly concordant (0.68–1.00; *p* < 0.001) and strongly opposed to hue angle (PCCH, −0.60 to −0.98); and in green coffee, a*, b*, and C* (GCCA/GCCB/GCCC) were positively correlated (0.22–1.00; *p* < 0.01) and opposed to hue (GCCH, −0.92 to 0.14).

Phenolic and antioxidant traits correlated with parchment and green coffee color. Total phenolics were positively associated (*p* < 0.01) with parchment a*, b*, and C* (TPC–PCCA, 0.30; TPC–PCCB, 0.22; TPC–PCCC, 0.22) and negatively with hue (TPC–PCCH, −0.28), while phenolics showed no significant associations with green coffee color (*p* > 0.05). ABTS and FRAP displayed significant relationships with color in both parchment and green coffee (ABTS–PCCA/PCCB/PCCC, 0.28–0.31; ABTS–GCCB/GCCC, 0.22; FRAP–PCCA/PCCB/PCCC, 0.22–0.29; FRAP–GCCB/GCCC, 0.20), whereas cherry color exhibited only very weak associations with bioactives.

Among secondary metabolites, chlorogenic acids were negatively associated (*p* < 0.05) with several cherry color parameters (CGA–CCCL, −0.25; CGA–CCCB, −0.18; CGA–CCCH, −0.21) and positively associated (*p* < 0.01) with green coffee a* (CGA–GCCA, 0.27). Trigonelline correlated with certain cherry color metrics (*p* < 0.05) (TGN–CCCA, 0.24; TGN–CCCB, 0.17; TGN–CCCH, −0.20), whereas caffeine showed only weak links with color parameters.

Fermentation variables aligned primarily with parchment coffee color. Initial and final soluble solids showed weak correlations with parchment and green coffee a*, b*, and C*. Initial pH (IPH) exhibited significant positive associations (*p* < 0.05) with parchment a* (0.41), b* (0.39), and C* (0.39), and a negative association with *h°* (−0.40); in green coffee, IPH also correlated significantly with a* (−0.18), b* (0.18), C* (0.18), and *h°* (0.27). By contrast, final pH (FPH) displayed significant inverse relationships (*p* < 0.05) with most chromaticity parameters of both parchment and green coffee. Collectively, Figure 2 indicates that bean color—particularly of parchment coffee—functions as an external proxy for phytochemical composition and antioxidant potential, whereas final fermentation pH modulates these chromatic attributes and associates directly with chlorogenic acids but inversely with flavonoids, caffeine, and ABTS.

### 2.5. Multivariate Analysis

To examine relationships among agro-morphological variables, bioactive compounds, antioxidant activity, and metabolites with respect to the region of origin, we generated scree plots and extracted variance-associated components. Additional details are provided in Supplementary Figures S1–S3.

**Figure 3:**
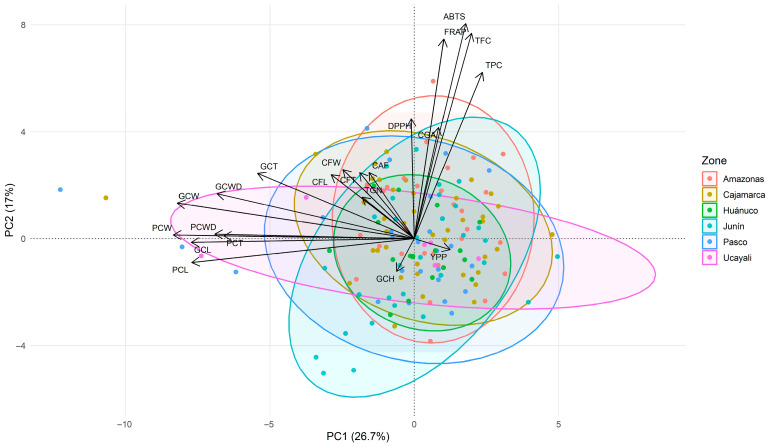
Principal component analysis (PCA) biplot showing the distribution of 150 coffee accessions based on agro-morphological parameters, bioactive compounds, and antioxidant activity, with accessions grouped by geographic origin. Abbreviations: FL, fruit length; FW, fruit width; FT, fruit thickness; PCW, parchment coffee weight; PCL, parchment coffee length; PCWD, parchment coffee width; PCT, parchment coffee thickness; GCW, green coffee weight; GCL, green coffee length; GCWD, green coffee width; GCT, green coffee thickness; GCH, green coffee moisture; YPP, yield per plant; TPC, total phenolic content; TFC, total flavonoid content; ABTS, DPPH, FRAP, antioxidant activity assays; TGN, trigonelline; CGA, chlorogenic acids; CAF, caffeine.

The first PCA biplot integrating bioactive/antioxidant and morphological traits revealed two main gradients (Figure 3). PC1 (26.7%) represented a morphometric axis, separating accessions with larger and heavier beans (negative PC1) from those with smaller seeds. PC2 (17.0%) captured a functional axis, with strong positive loadings for total phenolics, total flavonoids, and antioxidant activity, indicating higher bioactive levels in accessions with elevated PC2 scores. Green coffee moisture loaded in the opposite direction, while yield per plant contributed minimally to the first two components.

The secondary metabolites occupied intermediate positions in the biplot: chlorogenic acids clustered near the phenolic/antioxidant block, while caffeine and trigonelline showed moderate positive loadings on PC2. The overall biplot geometry indicated near-orthogonality between the morphometric and phenolic/antioxidant blocks, suggesting that bean size and antioxidant potential vary independently across the germplasm. Regional ellipses showed broad overlap, reflecting high intraregional heterogeneity and the absence of marked geographic segregation in the first two components.

**Figure 4:**
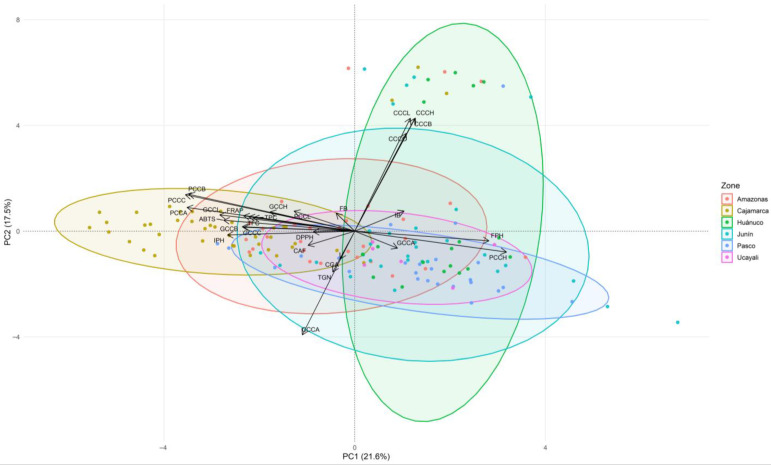
Principal component analysis (PCA) biplot showing the distribution of 150 coffee accessions based on color and fermentation parameters together with bioactive compounds and antioxidant activity, with accessions grouped by geographic origin. Abbreviations: CCCL, coffee cherry color L*; CCCA, coffee cherry color a*; CCCC, coffee cherry color C*; CCCB, coffee cherry color b*; CCCH, coffee cherry color *h°*; PCCL, parchment coffee color L*; PCCA, parchment coffee color a*; PCCC, parchment coffee color C*; PCCB, parchment coffee color b*; PCCH, parchment coffee color *h°*; GCCL, green coffee color L*; GCCA, green coffee color a*; GCCC, green coffee color C*; GCCB, green coffee color b*; GCCH, green coffee color *h°*; IB, initial soluble solids (°Brix); FB, final soluble solids (°Brix); IPH, initial pH; FPH, final pH; TPC, total phenolic content; TFC, total flavonoid content; ABTS, DPPH, FRAP, antioxidant activity assays; TGN, trigonelline; CGA, chlorogenic acids; CAF, caffeine.

The second PCA biplot, based on phytochemical, color (CIELAB), and fermentation variables, revealed two clearly defined gradients (Figure 4). PC1 (21.6%) separated accessions with greater color saturation in parchment and green coffee —aligned with higher levels of phenolic compounds and antioxidant activity (ABTS, FRAP, DPPH)— from those with higher hue angle (*h°*) and higher final pH after fermentation. PC2 (17.5%) was driven by cherry color: L*, b*, C*, and *h°* loaded upward, while a* loaded downward, distinguishing red from yellow cherries. Secondary metabolites occupied intermediate positions, with chlorogenic acids associated with the lower-left quadrant and caffeine and trigonelline showing shorter loadings and moderate associations.

The accession cloud is dense around the origin, yet regional ellipses suggest subtle geographic tendencies: Huánuco shows higher PC2 scores (clear differences in cherry color), Pasco shifts toward positive PC1 (higher final pH/*h°* in parchment coffee), and Cajamarca trends toward negative PC1 (greater color saturation aligned with higher phenolic/antioxidant content). Other regions largely overlap, indicating high intraregional heterogeneity. Taken together, this PCA indicates that postharvest color—particularly saturation in parchment and green beans—is associated with phenolic richness and antioxidant activity, whereas final pH and parchment hue angle (*h°*) oppose this profile; cherry color adds an independent dimension that helps explain additional variance among accessions.

**Figure 5:**
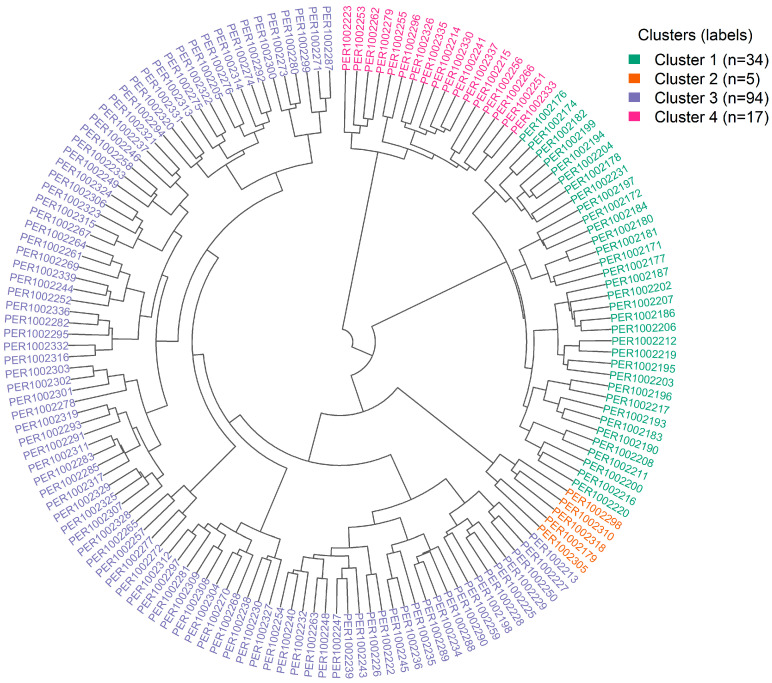
Circular dendrogram from hierarchical clustering of 150 coffee accessions based on agro-morphological, phytochemical, colorimetric, and fermentation parameters.

Hierarchical clustering was validated using the silhouette criterion (Supplementary Figure S4), which displayed a clear maximum in average silhouette width at k = 4, indicating that a four-cluster partition best balances intracluster compactness and intercluster separation in this multivariate space. The circular dendrogram in Figure 5 visualizes this Ward’s linkage (Euclidean distance) solution and reveals four topologically stable groups. Although accessions are color-coded by geographic origin, clusters comprise mixtures of regions, underscoring that phenotypic profiles—rather than provenance—govern cluster membership. Centroids derived from the multivariate variables indicate complementary trait constellations: Cluster 1 concentrates accessions with higher phenolics, flavonoids, and antioxidant responses (ABTS, DPPH, FRAP); Cluster 2 is characterized by larger bean dimensions and weights—a size-oriented phenotype with a tendency toward higher yield; Cluster 3 includes accessions with intermediate levels of bioactives but distinctive fermentation signatures (soluble solids and pH dynamics) and balanced yield; and Cluster 4 aggregates accessions defined by cherry CIELAB coordinates. The extensive overlap of regional colors within branches, together with the partial overlap of PCA confidence ellipses, confirms pronounced intraregional heterogeneity and the absence of strong geographic segregation in the first two components.

Table 2 summarizes the multivariate partition of the 150 accessions into four clusters and makes explicit, via accession codes (PER codes), each cluster’s distribution by region of origin. The partition is markedly uneven: Cluster 1 comprises 34 accessions (22.7% of the collection), Cluster 2 is a small, specialized set of 5 accessions (3.3%), Cluster 3 is the largest with 94 (62.7%), and Cluster 4 includes 17 accessions (11.3%).

Cluster 1 is distinguished by elevated levels of bioactive compounds—high total phenolics, total flavonoids, and antioxidant activity. It is dominated by materials from Cajamarca (33 of 34 accessions), with a single accession from Amazonas (PER1002231), underscoring that this functional phenotype is not exclusive to a single provenance.

Cluster 2 is a small group defined by agro-morphological variables; it contains five accessions: one from Cajamarca (PER1002179), three from Pasco (PER1002298, PER1002305, PER1002310), and one from Ucayali (PER1002318).

Cluster 3 constitutes the central diversity group, combining intermediate-to-favorable yield per plant with moderate levels of bioactives and distinctive fermentation signatures. It is also the most geographically diverse: four accessions from Cajamarca, 25 from Amazonas, 26 from Junín, 21 from Pasco, 17 from Huánuco, and one from Ucayali. The breadth of this cluster confirms pronounced intraregional heterogeneity and provides the widest genetic base for multi-trait selection.

Cluster 4 comprises 17 accessions defined by cherry color coordinates: three from Cajamarca, three from Amazonas, five from Junín, five from Huánuco, and one from Pasco.

Taken together, the sum across clusters yields a global regional contribution of 41 accessions from Cajamarca, 31 from Junín, 29 from Amazonas, 25 from Pasco, 22 from Huánuco, and two from Ucayali. Notably, every region is represented in at least one cluster—and most in several—indicating that provenance alone does not predict cluster membership. Practically, these results enable goal-oriented sampling: Cajamarca constitutes the principal source of antioxidant-rich genotypes in Cluster 1; Pasco contributes prominently to Cluster 2, which is oriented toward bean size and agro-morphological traits; all regions feed the diverse Cluster 3 with balanced ideotypes and fermentation traits; and Junín–Huánuco concentrate the uncommon chromatic profiles that define Cluster 4.

**Figure 6:**
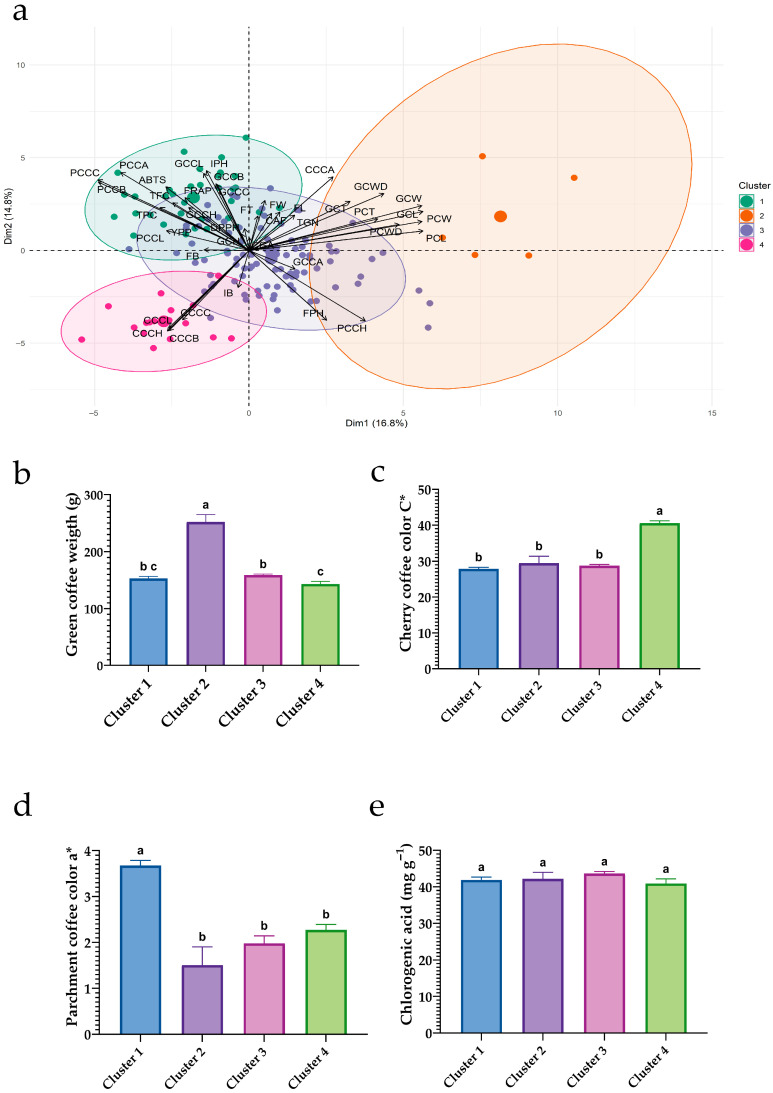
Multivariate clustering and contrasts of key traits among clusters in 150 coffee accessions. (**a**) PCA biplot constructed from morphological, color, fermentation, and bioactive variables; ellipses denote the four clusters obtained via hierarchical clustering (Ward–Euclidean). (**b**) Green bean weight by cluster; (**c**) cherry chroma (C*) by cluster; (**d**) parchment coffee red–green coordinate (a*) by cluster; (**e**) chlorogenic acid content (mg g^−1^) by cluster. Bar charts (**b**–**e**) show means ± SEM; different letters above bars indicate significant differences among clusters (Tukey HSD, *p* < 0.05).

Figure 6 highlights four complementary phenotypic profiles: (i) Cluster 2—a morphometric/size profile with heavier beans; (ii) Cluster 4—a profile with high cherry color intensity (elevated C*); (iii) Cluster 1—a quality-oriented profile aligned with the phenolic–antioxidant axis; and (iv) Cluster 3—an intermediate, balanced ideotype. These contrasts provide a clear basis for selection depending on whether the priority is bean size, fruit color intensity, or functional attributes (phenolics and antioxidants).

### 2.6. Promising Coffee Accessions

**Figure 7:**
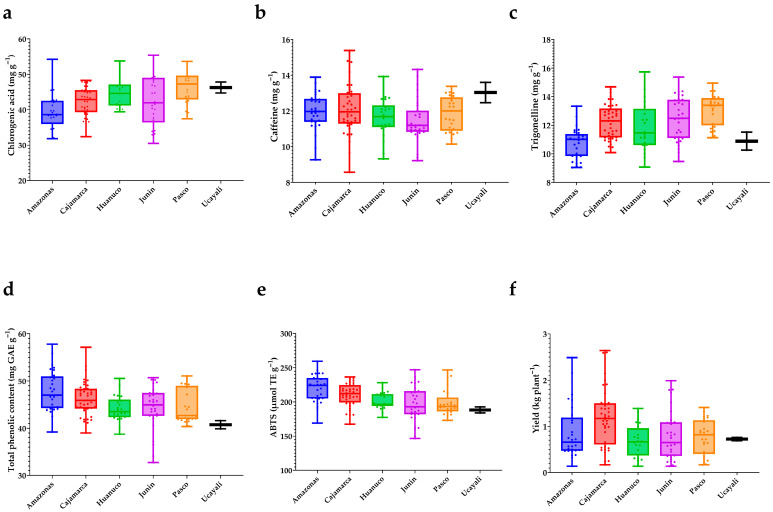
Variation in bioactive compounds, antioxidant activity, and yield among Peruvian *Coffea arabica* L. accessions by region of origin. (**a**) Chlorogenic acid (mg g^−1^); (**b**) caffeine (mg g^−1^); (**c**) trigonelline (mg g^−1^); (**d**) total phenolic content (mg GAE g^−1^); (**e**) antioxidant activity by ABTS (μmol TE g^−1^); (**f**) yield (kg plant^−1^). Boxplots display the full range with median lines; whiskers denote minimum and maximum values; points represent individual accessions.

In Figure 7a–f summarize the distribution of key traits by geographic origin. For chlorogenic acids (a), medians are highest in Pasco and Ucayali, intermediate in Huánuco and Cajamarca, and lowest in Amazonas and Junín; dispersion is widest in Junín and narrowest in Ucayali, indicating both regional contrasts and substantial within-region variation. Caffeine (b) exhibits a different pattern: Cajamarca and Junín display the broadest spreads, whereas Amazonas, Pasco, Huánuco, and Ucayali occupy intermediate positions with overlapping interquartile ranges. For trigonelline (c), Junín and Pasco show higher medians, Cajamarca and Huánuco are intermediate, and Amazonas and Ucayali lie at the lower end.

The phenolic/antioxidant block reveals coherent trends. Total phenolic content (d) is elevated in Amazonas and Cajamarca, intermediate in Junín and Huánuco, and lower in Pasco and Ucayali; Huánuco shows moderate dispersion with a mid-low median. ABTS antioxidant activity (e) mirrors the phenolic pattern, with higher medians in Amazonas and Cajamarca, lower values in Ucayali, and the remaining regions clustered in intermediate positions. Finally, yield per plant (f) exhibits the greatest relative dispersion among traits: Cajamarca shows the highest central tendency and upper extremes, Ucayali trends lower, and Amazonas displays a wide spread, underscoring pronounced intraregional heterogeneity. Collectively, the boxplots indicate significant regional tendencies while revealing substantial overlap of interquartile ranges, confirming that although origin shapes central tendencies, within-region variability remains high and outstanding profiles—whether in bioactive content, antioxidant activity, or agronomic performance—are not confined to any single region.

**Figure 8:**
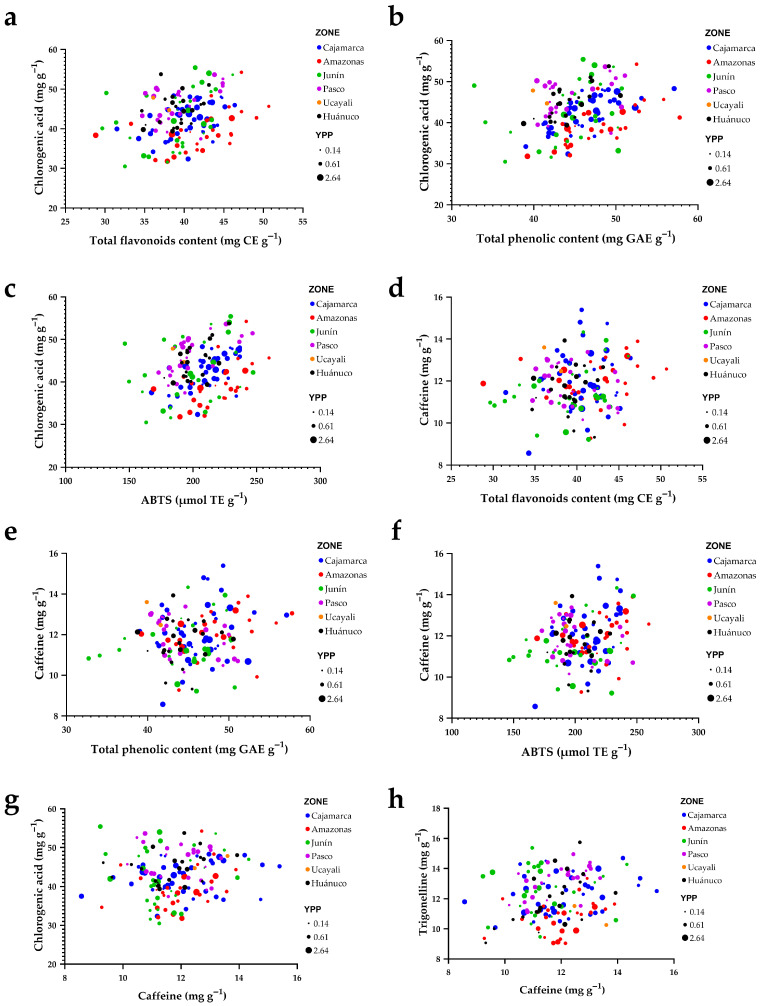
Bivariate relationships among bioactive compounds, antioxidant activity, and caffeine by region of origin. (**a**) Chlorogenic acids vs. total flavonoids; (**b**) chlorogenic acids vs. total phenolics; (**c**) chlorogenic acids vs. ABTS antioxidant activity; (**d**) caffeine vs. total flavonoids; (**e**) caffeine vs. total phenolics; (**f**) caffeine vs. ABTS antioxidant activity; (**g**) chlorogenic acids vs. caffeine; (**h**) trigonelline vs. caffeine. Points are colored by region and scaled by yield per plant (YPP).

Figure 8 integrates relationships among key phytochemical and antioxidant metrics. Along the phenolic gradient, chlorogenic acids (CGA) increase systematically with total flavonoids and total phenolics (panels a,b) and also rise with ABTS antioxidant activity (panel c). The upper-right region of these plots concentrates accessions that simultaneously exhibit high CGA, elevated phenolics/flavonoids, and strong ABTS responses, marking them as candidates for functional quality. Regional origins (colors) are extensively intermingled along these trends, indicating that the chemical synergy between the phenolic pool and radical-scavenging capacity occurs across regions rather than being restricted to any single provenance.

By contrast, caffeine behaves largely as an independent axis of variation. Its point clouds against flavonoids, total phenolics, and ABTS (panels d–f) are diffuse and show no clear trend, implying weak association with the phenolic–antioxidant block. Comparing alkaloids, the CGA–caffeine relationship (panel g) suggests a slight inverse tendency, whereas trigonelline increases with caffeine (panel h), revealing coordinated accumulation for some metabolites.

Across all panels, point size (yield per plant) spans the full range at both low and high metabolite/antioxidant values, indicating no evident trade-off between yield and phytochemical traits. Collectively, Figure 8 suggests that strengthening phenolics and flavonoids provides a direct route to increasing CGA and ABTS activity, whereas caffeine can be modulated in a semi-independent manner—an advantageous scenario for breeding programs aiming to combine strong antioxidant potential with balanced caffeine levels without sacrificing agronomic performance.

**Figure 9:**
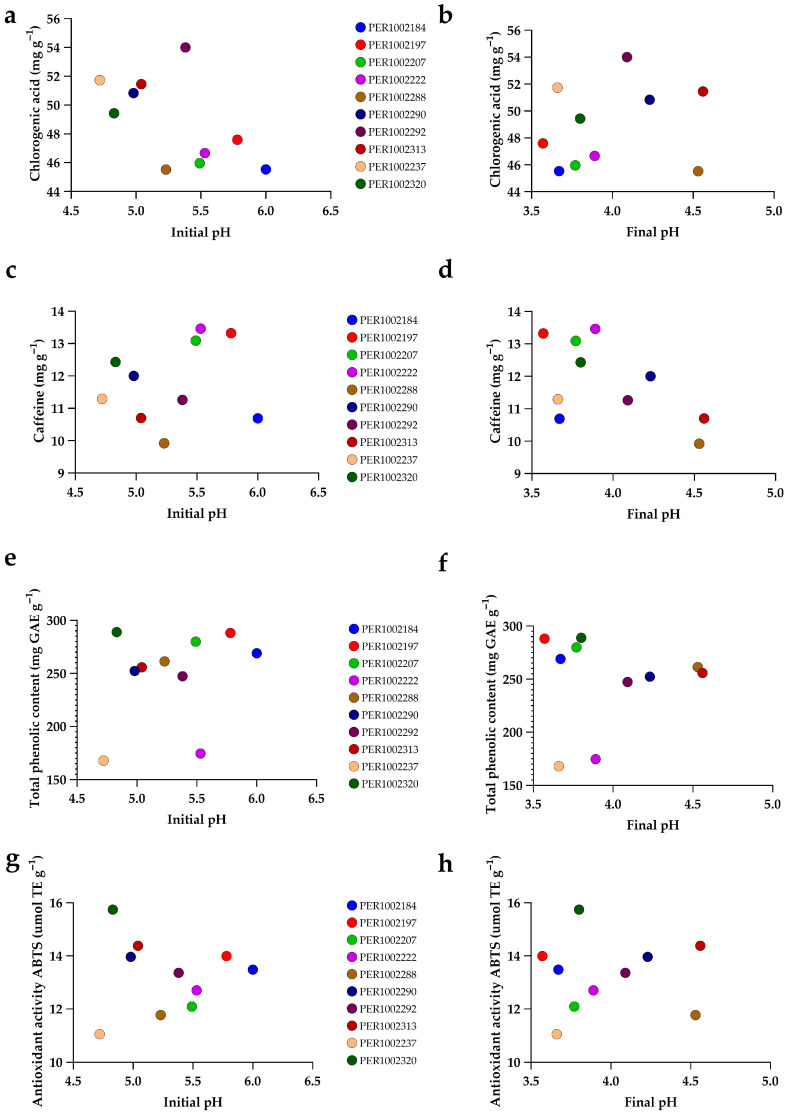
Relationships between fermentation pH and bioactive/antioxidant traits in the top 10 selected coffee accessions. (**a**,**b**) Chlorogenic acids (mg g^−1^) vs. initial pH and final pH; (**c**,**d**) caffeine (mg g^−1^) vs. initial pH and final pH; (**e**,**f**) total phenolic content (mg GAE g^−1^) vs. initial pH and final pH; (**g**,**h**) ABTS antioxidant activity (μmol TE g^−1^) vs. initial pH and final pH. Each point represents an accession (IDs in the legend; colors are consistent across panels). pH was recorded at the beginning and at the end of fermentation for the same samples.

Figure 9 examines how initial and final fermentation pH relate to key phytochemical traits in a representative subset of accessions (IDs in the legend). Two consistent patterns emerge. First, final pH shows a slight positive tendency with chlorogenic acid (panel b), whereas no clear trend is apparent with caffeine (d), total phenolics (f), or ABTS (h). Accessions that finish fermentation at higher pH (4.0–4.6) cluster toward higher CGA (49–52 mg g^−1^), caffeine (10.7–12.5 mg g^−1^), total phenolics (47–51.2 mg GAE g^−1^), and ABTS (227–247 μmol TE g^−1^), while those with lower final pH tend to exhibit lower values for these traits. Second, initial pH shows weak associations: CGA and total phenolics (a, e) are largely horizontal clouds, indicating little systematic variation over the observed IPH range (4.7–6.0); caffeine vs. initial pH (c) is diffuse and non-linear; and ABTS vs. initial pH (g) displays modest dispersion around a flat trend. Taken together, these panels indicate that the fermentation endpoint (final pH) is more informative than initial pH for the evaluated phytochemical status in this subset. Notably, these patterns are subset-specific: while the full-dataset correlation matrix (Figure 2) showed generally inverse global associations between final pH and the phenolic/antioxidant block, the present figure suggests that within this group, accessions with less acidified fermentations (higher final pH) tend to show lower phenolics, caffeine, and ABTS but a direct relationship with CGA. This reinforces the idea that fermentation dynamics can predict chemical outcomes at the accession level.

The top tier comprised ten promising accessions spanning five Peruvian regions (Pasco, Cajamarca, Junín, Huánuco, and Amazonas), underscoring that outstanding profiles are geographically distributed rather than confined to a single origin. The highest overall score (Supplementary Table S1) was recorded for PER1002197 (Cajamarca; 48/50), which combined top individual scores for chlorogenic acids, phenolics, flavonoids, ABTS, and bean size/weight, together with high caffeine content. PER1002222 (Cajamarca; 46/50) likewise paired elevated caffeine with strong phenolic/antioxidant metrics and robust bean morphometry. PER1002207 (Cajamarca; 45/50) also exhibited a balanced profile, achieving maximum scores in yield, bean weight, and dimensions, in addition to high flavonoids and caffeine. PER1002288 (Pasco; 42/50) stood out for antioxidant/phenolic values, with comparatively lower caffeine but high CGA. PER1002292 (Pasco; 42/50) combined outstanding CGA and trigonelline with consistent antioxidant metrics and favorable bean size.

The next tier shares comparable overall scores but differs in specific selection criteria. PER1002184 (Cajamarca; 41/50) combined high chlorogenic acids and trigonelline with low caffeine and moderate bean size. PER1002313 (Junín; 41/50), PER1002290 (Pasco; 41/50), PER1002320 (Huánuco; 41/50), and PER1002237 (Amazonas; 41/50) exhibited intermediate scores (2–5) for phenolics, flavonoids, and ABTS, coupled with superior bean size.

## 3. Discussion

Significant differences were detected among the six geographic regions of origin represented in the INIA National Collection with respect to morphological traits, phytochemical composition, and antioxidant activity. Morphologically, accessions varied markedly in fruit and bean form. Accessions with lower yield tended to exhibit greater parchment and green bean weight and dimensions, yielding a weak negative correlation between production and bean size metrics. This pattern is consistent with Leon-Burgos et al. [32], who reported that coffee plants bearing a high fruit load produce a smaller proportion of large beans. Thus, yield per plant and parchment bean dimensions are partially decoupled; however, this does not imply that lots with larger beans but lower yield per plant necessarily exceed, in final mass, standard-sized beans produced at higher yield.

With respect to the interaction between ecological factors and cultivation, the environment can shape productivity and, in turn, drive variation in bean quality. Prior studies show that shade conditions affect flowering, an intrinsically light-dependent stage, often resulting in reduced production [33,34]. Nonetheless, shade can slow fruit maturation and favor larger bean size and development, potentially enhancing certain quality attributes [34]. This adaptive response to environmental conditions is also genotype-dependent [35]. In germplasm grown under shade-tree associations, both production and bean size may be influenced by shade; however, responses to these conditions vary by genotype. Yield and bean size thus serve as key indicators of productivity and market quality. In our dataset, yield per plant (YPP) was not significantly correlated with coffee bean dimensions, a result consistent with studies showing that high fruit load reduces the proportion of large, healthy beans due to resource competition [32]. Consequently, within the Peruvian germplasm analyzed, selection on yield alone does not necessarily improve the physical attributes of the bean, underscoring the need to integrate morphological and productivity criteria as partially independent targets in breeding programs. At the same time, the regional contrasts observed—particularly the larger bean weight and dimensions in accessions from Cajamarca and Pasco—offer valuable material for breeding strategies seeking to enhance YPP while safeguarding grain size. As noted by Ngure and Watanabe [36], the continued adoption of improved varieties adapted to site-specific conditions is critical to securing the future of coffee.

Green coffee moisture (GCH) was positively associated with physical dimensions (weight, length, width, and thickness), consistent with reports in both robusta and arabica indicating that moisture influences bean size and density [37,38]. Likewise, Worku et al. [39] and Wale et al. [40] show that altitude and processing conditions affect bean weight, density, and defect rates, confirming that physical quality and yield respond to the interaction between environment and postharvest management. From a safety standpoint, appropriate bean moisture is essential to prevent conditions favorable to toxigenic fungi and the formation of mycotoxins such as ochratoxin A [41]. Although roasting can reduce toxin levels, it does not guarantee complete elimination, underscoring the need to prevent contamination from the earliest stages of processing [42].

The results reveal pronounced variability in phytochemical traits among the 150 Peruvian *C. arabica* accessions, reflecting the influence of both genetic factors and environmental conditions. This agrees with Urugo et al. [43] who reported origin-dependent variation in Ethiopian coffees for major compounds such as caffeine (0.88–1.07% dry weight) and chlorogenic acids (3.7–8.2%). In our study, accessions from higher-altitude origins generally exhibited superior quality metrics: many highland coffees showed elevated total phenolics and greater antioxidant activity, together with denser, larger beans [39,44,45,46]. This pattern aligns with reports that cooler high-elevation climates prolong bean maturation, favoring improved development and the accumulation of polyphenols [47,48]. Recent work on altitude effects similarly indicates that lowland beans tend to have lower total phenolics, whereas high-elevation beans are richer in phenolics (and associated volatiles) due to cooler growth conditions [47,49,50,51]. In our low-altitude accessions, we observed a tendency toward slightly higher caffeine content, consistent with Chen [52], who found that increasing altitude is associated with declines in caffeine and 5-CQA. This altitude–caffeine relationship is physiologically plausible, as plants at lower, warmer sites often produce more caffeine as a defensive compound [53,54]. Nevertheless, altitude effects are not absolute; for example, an Ethiopian study reported the highest caffeine levels in a highland region (Yirgacheffe) and the lowest in a mid-altitude region (Harar), suggesting that local varietal genetics and microclimates can modulate—or even counteract—general altitudinal trends [55]. In concordance, our findings indicate that coffee phytochemical profiles respond to a complex interaction between regional environment and cultivar genotype [17,56]. Thus, even when grown at the same elevation (774 m a.s.l.), accessions retain, to some extent, genetic signatures linked to provenance that shape their productive and biochemical profiles. This underscores the role of germplasm banks as repositories of genetic and phytochemical diversity, essential for breeding and for enhancing the resilience of coffee systems under climate change. Although the multivariate results showed certain tendencies associated with the geographical origin of the accessions, these should be interpreted with caution. Since all accessions were cultivated under the same environmental conditions in Pichanaki, the observed differences mainly reflect the phenotypic expression of genetic variability in a common environment rather than environmental effects associated with their original collection sites.

Genetic diversity among these Peruvian accessions contributed to the wide variation observed in bioactive compounds. Nearly two-fold differences were detected between the minimum and maximum values for total phenolic content (TPC) and total flavonoid content (TFC), with an even broader range for caffeine. A study on green coffee beans reported average values of 25.1 mg GAE g^−1^ for total phenolics and 8.19 mg g^−1^ for chlorogenic acids, both below the levels recorded in the present study. In contrast, caffeine content (12.6 mg g^−1^) remained within the range observed in our *Coffea arabica* accessions [57]. Similarly, Hameed et al. [58] documented that caffeine concentration can vary by approximately threefold among *C. arabica* genotypes (0.6–1.8% dry matter), underscoring the importance of genetic constitution. Several traditionally cultivated accessions (analogous to Typica/Bourbon lines) exhibited intrinsically higher TPC and antioxidant readouts than others, suggesting an inherent varietal capacity for secondary metabolite biosynthesis [44,59]. Conversely, certain high-yielding, disease-resistant varieties (Catimor-type hybrids) have been reported to display relatively lower TPC or antioxidant activity, reflecting trade-offs noted in coffee breeding programs [60,61]. Indeed, genotype–environment interaction emerged clearly in our PCA and clustering analyses. The PCA biplot indicated that the first principal component was strongly influenced by phytochemical traits (phenolics, flavonoids, and DPPH/ABTS/FRAP assays), separating accessions with superior nutraceutical profiles from those with lower values. The second component appeared to capture variation in agronomic traits (yield, fruit size, bean size, etc.), distinguishing high-yielding accessions with comparatively lower phenolics from lower-yielding accessions with richer phytochemical composition. Hierarchical clustering further reinforced these patterns: accessions grouped by multi-trait profiles that frequently aligned with region of origin or varietal group. In this study, many higher-elevation accessions from the Selva Central clustered together, characterized by above-average antioxidant activity and larger beans, whereas a cluster of lower-elevation accessions from the northern coast shared traits such as smaller beans, higher caffeine, and lower TPC. Such groupings echo the multifactorial patterns reported by Makiso et al. [62], where geographic and climatic variables explained ~60% of the variation in bean properties and chemistry in Ethiopia. Our findings reinforce that both environmental terroir and genetic background jointly determine coffee quality traits, and that integrated multivariate analysis is a powerful approach to disentangle these effects [17,56].

Strong correlations were observed among phytochemical traits in our accessions. Total phenolic content (TPC) showed a highly positive association (*p* < 0.001) with ABTS (0.66,) and FRAP (0.51). These results agree with previous findings in coffee beans, where similarly strong correlations (*p* < 0.01) were reported for TPC-ABTS (0.77) and TPC-DPPH (0.64) [63]. Likewise, Wu et al. [64] reported significant correlations (*p* < 0.01) between phenolic compounds and antioxidant activity (TPC–ABTS, 0.75; TPC–DPPH, 0.79; TPC–FRAP, 0.79), values comparable to those obtained in the present study. These findings indicate that phenolics are the main contributors to radical-scavenging capacity in these coffees [65,66]. This aligns with numerous reports linking higher polyphenol levels to greater antioxidant power in green coffee extracts [67,68]. In our data, accessions with higher total phenolic content (TPC) and total flavonoid content (TFC) exhibited elevated ABTS and FRAP values, whereas TFC showed only moderate associations with DPPH, underscoring that phenolics—particularly chlorogenic acids, which correlated positively with ABTS (0.24, *p* < 0.01) and FRAP (0.21, *p* < 0.01)— are the main drivers of antioxidant potential [8,69]. By contrast, caffeine content displayed minimal correlation with antioxidant metrics, as expected for an alkaloid with comparatively lower activity than phenolics; this is consistent with Sualeh et al. [7], who reported a modest correlation between caffeine and DPPH (0.18).

Caffeine showed a low positive correlation with TPC, suggesting that beans with higher phenolic concentration often contain moderate caffeine levels—a favorable combination from a health perspective [9,70]. Similar inverse tendencies have been reported when examining altitude effects (higher phenolics with lower caffeine at greater elevations) [45,71]. It is also noteworthy that chlorogenic acids (CGAs)—the dominant subclass of phenolics in green coffee—largely underpin coffee’s antioxidant effects and health benefits [72,73]. In these Peruvian accessions, CGAs likely constituted a substantial proportion of total phenolics and are recognized both for conferring antioxidant activity and for influencing cup quality [72,74,75]. Several of our high-TPC accessions presumably had elevated CGA content, which can be advantageous nutraceutically, although excessively high levels may pose sensory challenges [69,76]. The literature remains divided on the flavor impact of CGAs: while they are antioxidant, very high CGA concentrations have been associated with increased astringency and “off-flavors” in the cup [76,77,78]. Our discussion acknowledges this balance: breeding aimed at enhancing polyphenols should also consider cup quality. Moderate increases in CGAs and related phenolics can improve coffee’s health profile, whereas excessive levels may detract from flavor—particularly because CGA degradation during roasting contributes to bitterness [69,79]. Fortunately, roasting attenuates a substantial fraction of CGAs, suggesting that observed levels can be managed within acceptable ranges to maintain quality. In addition, we quantified other bioactives such as trigonelline, which—although not correlated with total phenolic content (TPC)—is an important alkaloid that degrades during roasting into compounds contributing to desirable flavor, aroma, and antioxidant activity [80,81]. However, in the green coffee beans analyzed in this study, trigonelline showed no significant association with any of the antioxidant assays, which is consistent with its limited redox reactivity in the unroasted state. This pattern parallels that of caffeine, as both alkaloids originate from nitrogen-based pathways (purine or pyridine nucleotide) rather than from the phenylpropanoid route of phenolics [82], which explains their weak redox activity and lack of association with antioxidant activity.

Within a breeding-oriented framework, we developed a selection index to identify the most promising accessions based on an integrated profile combining yield, physical bean quality, and nutraceutical value. Each accession was scored from 1 (poor) to 5 (excellent) across ten key variables: yield per plant, green-bean size (caliber) and weight, total phenolics, total flavonoids, ABTS antioxidant activity, chlorogenic acids, trigonelline, and caffeine. This scoring scheme enabled an objective, multi-trait ranking of accessions. Notably, a subset achieved high scores (4–5) in nearly all categories, emerging as top performers. These accessions exhibited high productivity (often >30% above the trial mean) alongside outstanding phytochemical profiles. Among first-tier materials from Cajamarca, PER1002197 and PER1002222 combined strong yield and competitive bean size with elevated phenolics, robust antioxidant activity, favorable chlorogenic acid and trigonelline levels, and balanced caffeine; meanwhile, PER1002288 (Pasco) and PER1002184 (Cajamarca) showed similarly favorable profiles but with lower caffeine content.

These genotypes are of substantial value: they demonstrate that yield and nutraceutical quality need not be mutually exclusive. Indeed, the correlation analysis found no inherent antagonism between yield and polyphenol content, a crucial finding for breeding [83,84]. The top-performing accessions identified here can be leveraged in improvement programs either as parents or as new cultivars for growers. Through crossing or clonal propagation of these superior types, breeders can develop *C. arabica* varieties that deliver strong agronomic returns to farmers while offering consumers specialty coffees with added health benefits.

Accessions with high caffeine content also represent a valuable resource. Beyond its stimulant effect, caffeine has been associated with physiological benefits such as improved attention and cognitive performance, increased physical capacity, and modulation of energy metabolism, in addition to contributing to antioxidant properties [85,86]. These attributes largely explain the global preference for coffees with higher caffeine concentration, particularly in markets seeking natural energizing beverages or differentiated sensory experiences [7]. At the same time, accessions with naturally low caffeine levels are strategically important, as they open avenues to develop coffees with reduced caffeine without resorting to decaffeination processes. In line with consumption trends, the global decaffeinated coffee market reached USD 2.39 billion in 2024 and is projected to grow at 5.3% annually to USD 3.28 billion by 2030 [87]. Given that average caffeine in *C. arabica* is ~1.2%, the discovery of accessions near 0.8% or lower (as observed in this study) is particularly relevant [61]. Hameed et al. [58], emphasized the scarcity of low-caffeine arabica in the genetic pool; thus, the identification of multiple low-caffeine accessions with strong phytochemical profiles constitutes a promising opportunity to diversify coffee products for caffeine-sensitive consumers. In this sense, the coexistence of both low- and high-caffeine accessions within the germplasm collection is a strategically diverse asset, expanding breeding possibilities and enabling responses to distinct consumer niches in a continually diversifying global market.

From a nutraceutical perspective, the implications of these findings are substantial. Coffee is increasingly recognized not only as a stimulant beverage but also as a functional food with potential health benefits [88]. The high antioxidant levels (phenolics and flavonoids) measured in our top accessions suggest that these coffees could deliver enhanced health value. Antioxidant assays (DPPH, ABTS, FRAP) in the best-performing accessions were comparable to, or exceeded, values reported for other high-antioxidant coffees, indicating strong free-radical–scavenging capacity [66,89]. Each quantified bioactive—caffeine, chlorogenic acids, and trigonelline—plays a role in human health. Although often considered solely a stimulant, caffeine has shown context-dependent protective effects, including anticancer activity and hepatoprotection [90]. Chlorogenic acids are well-documented antioxidants linked to reduced risk of type 2 diabetes and cardiovascular disease [78,91]. Trigonelline has been shown to modulate oxidative stress and may help prevent kidney stone formation [92]. In light of these benefits, enhancing these compounds in coffee beans is a desirable breeding objective. Our results indicate that simultaneous selection for high CGAs, trigonelline, and TPC is feasible—traits that could render a coffee genuinely “cardio-healthy” or “rich in antioxidants.” If such coffees reach the market, they could be positioned with a wellness focus, potentially commanding competitive price premiums [67,71,83]. Moreover, moderate consumption of polyphenol-rich coffee has been associated with lower risk of several chronic diseases; thus, cultivating varieties with elevated nutraceutical content aligns with public-health trends. We also foresee applications beyond the cup: green coffees with high phenolic content could serve as excellent sources for extracting natural antioxidants or producing phenolic supplements. In this context, green coffee bean extracts (GCBE) derived from the top accessions identified here may constitute promising nutraceutical products, leveraging their elevated total phenolic and flavonoid content.

From a palatability standpoint, it is essential to balance quality with sensory appeal when leveraging these traits. Although the promising accessions reported here meet agronomic and nutraceutical performance criteria, they must also satisfy specialty-coffee sensory expectations. Notably, many health-beneficial phytochemicals also shape flavor. Higher acidity (lower pH) and greater sugar content (°Brix) in the coffee fruit—assessed here as fermentation metrics—can translate into a brighter, sweeter cup [93,94]. We observed some variation among accessions in mucilage soluble solids and pH, although these parameters carried less weight in the selection index. Even so, accessions with lower end-of-fermentation mucilage pH—indicative of higher organic-acid levels—tended to correspond to high-elevation coffees known for their acidity. Altitude is again relevant: high-elevation beans are often valued for their acidity and flavor complexity, and our results suggest that accessions from the highest sites (Junín and Cajamarca) exhibited slightly lower mucilage pH (more acidic) and, consequently, stronger performance in the selection index [53,95]. This is consistent with the general observation that high-altitude coffees develop greater acidity and organoleptic complexity, contributing to premium status. Shade-grown conditions and longer maturation at higher elevations may also increase sugar accumulation in cherries, potentially enhancing cup sweetness [96,97], factors that integrate into holistic coffee quality. Importantly, several top accessions in this study appear to achieve an outstanding balance: despite thriving at relatively low elevation (774 m a.s.l.), they possess genetics tuned for quality, accumulate health-beneficial compounds, and do so without compromising cup attributes. Early SCAA cuppings of leading accessions (e.g., PER1002197) revealed clean, complex cups with above-average fragrance and acidity [98], an encouraging indication that the reported promising accessions can also perform as specialty coffees (personal communication). Although this study was not designed as an applied breeding trial, the integrated evaluation of agronomic, physicochemical, and phytochemical traits provides a valuable baseline for future selection and pre-breeding initiatives within the Peruvian *Coffea arabica* germplasm.

## 4. Conclusions

This study integrates—at a novel, globally unprecedented scale for coffee—agro-morphological, colorimetric, fermentation, and phytochemical characterization of Peruvian *Coffea arabica* L. germplasm, and demonstrates that the available phenotypic diversity permits the combination of yield and nutraceutical quality without severe trade-offs. Correlation and PCA analyses resolved two nearly orthogonal axes—one morphometric (bean weight and dimensions) and one functional (phenolics, total flavonoids, chlorogenic acids, and antioxidant activity by ABTS/DPPH/FRAP)—indicating that increasing bean size can be achieved semi-independently of strengthening the phytochemical/antioxidant profile. Postharvest color parameters, particularly parchment and green-bean chroma, associated positively with phenolic richness and antioxidant potential, whereas final fermentation pH showed opposite relationships; together, these variables emerge as operational indicators of chemical quality. Hierarchical clustering (k = 4) revealed complementary ideotypes—size-oriented, phenolic–antioxidant–oriented, fruit-color–oriented, and balanced profiles—with broad geographic overlap, underscoring that provenance alone does not determine multi-trait performance. Using a 10-variable selection index (1–5 scale), we identified elite accessions—among them PER1002197, PER1002222, PER1002288, and PER1002184—that combine competitive bean caliber and yield with high chlorogenic acids, phenolics, and flavonoids, strong antioxidant activity, and, in some cases, naturally high or low caffeine—an ideal profile for specialty markets and nutraceutical applications. Collectively, these findings provide a robust empirical basis to guide crossing and assisted selection toward “climate-smart” and “health-smart” varieties capable of sustaining productivity while differentiating on functional value, and they establish practical metrics (CIELAB color and fermentation pH) for evaluation and quality control along the postharvest chain.

## 5. Materials and Methods

### 5.1. Plant Material

**Figure 10:**
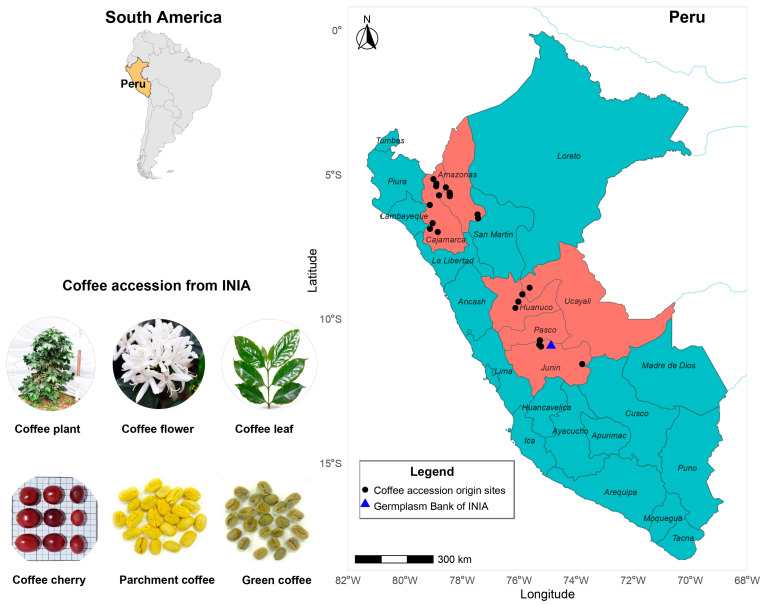
Collection zones of the accessions and location of the INIA *Coffea arabica* L. germplasm collection (Peru).

The National *Coffea arabica* L. Germplasm Collection is established at the Pichanaki Agricultural Experiment Station, Junín, Peru, at 774 m a.s.l. (10°55′29″ S, 74°52′36″ W). The collection comprises 169 accessions gathered from Peru’s principal coffee-growing regions (Cajamarca, Amazonas, Huánuco, Junín, Pasco, and Ucayali; see Figure 10). For this study, 150 accessions were evaluated (Supplementary Table S2). Nineteen accessions were excluded due to intra-accession phenotypic heterogeneity, evidenced by morphological differences among plants belonging to the same accession. Plants were grown at 2.5 × 1.0 m spacing, with 10 plants per accession, of which five plants were evaluated for the collection of ripe fruits. The germplasm is maintained under a native forest-tree association; site conditions include a mean temperature of 26.50 °C, relative humidity of 74.41%, and mean annual precipitation of 1625.4 mm.

The evaluation was conducted during a single coffee production cycle, beginning with the flowering period in June 2022 and extending until July 2023. This period corresponds to the 2022–2023 coffee harvest season, according to the local agricultural calendar. Harvest and postharvest processing followed the wet method (selection, depulping, fermentation, and drying) in accordance with guidelines from Romero and Camilo [99], the National Institute for Quality [100] and INIA [101]. Fermentation was carried out naturally for 12–16 h under the ambient conditions of the EEA Pichanaki. Physicochemical characterization was performed at the Laboratorio de Investigación Nutricional de los Recursos Genéticos at INIA’s central headquarters.

### 5.2. Reagents, Chemicals, and Sample Preparation

Analytical-grade reagents included Folin–Ciocalteu reagent, sodium carbonate, sodium nitrite, aluminum chloride, sodium hydroxide, potassium persulfate, ferric chloride, sodium acetate, ferrous sulfate, glacial acetic acid, 2,2-diphenyl-1-picrylhydrazyl (DPPH), 2,2′-azino-bis(3-ethylbenzothiazoline-6-sulfonic acid) diammonium salt (ABTS), 2,4,6-tripyridyl-s-triazine (TPTZ), and HPLC-grade solvents (methanol, ethanol, acetonitrile). All were procured from Merck (Darmstadt, Germany), Sigma-Aldrich (St. Louis, MO, USA), and J.T. Baker (Phillipsburg, NJ, USA). Standards were obtained from Merck (Darmstadt, Germany), Supelco (Bellefonte, PA, USA), Chem-Impex (Wood Dale, IL, USA), and Sigma-Aldrich (St. Louis, MO, USA). Ultrapure water was produced with a Micro-Pure ST purification system from Thermo Scientific (Waltham, MA, USA).

For the determination of bioactive compounds, antioxidant activity, and the quantification of secondary metabolites, green coffee beans were milled in an ultracentrifugal mill (Retsch ZM 200, Retsch, Haan, Germany) at 18,000 rpm until a particle size smaller than 0.5 mm (φ < 0.5 mm) was obtained. The ground material was stored in airtight containers, protected from light and moisture.

### 5.3. Yield per Plant

Yield per plant (kg plant^−1^) was determined following IPGRI guidelines [102]. Ripe fruits from each plant were harvested throughout the season, and yield was expressed as the cumulative mass of fruits collected per plant.

### 5.4. Fermentation Parameters

Soluble solids (°Brix) were quantified according to ISO 2173 [103], with minor modifications. Natural fermentation was carried out immediately after depulping the coffee cherries. For the determination of soluble solids, an aliquot of the mucilage mixture was extracted and measured using a refractometer (Hanna HI96811, Hanna Instruments, Woonsocket, RI, USA). The pH was measured following ISO 1842 [104], with modifications; measurements were performed with a potentiometer (Oakton 2700, Oakton Instruments, Vernon Hills, IL, USA) by inserting the electrode directly into the mucilage. All measurements were performed in triplicate. Both variables were evaluated at the beginning and at the end of the fermentation process.

### 5.5. Morphological and Color Characterization of Coffee

#### 5.5.1. Morphological Parameters

Morphological measurements followed the descriptors in IPGRI [102]. Five coffee fruits, five parchment beans, and five green beans representatives per accession were selected to measure length, width, and thickness using a digital caliper (Mitutoyo CD-6” ASX-B, Mitutoyo Corp., Kawasaki, Japan). Parchment and green-bean weights were determined with an analytical balance.

#### 5.5.2. Color Analysis

Color (CIELAB) was measured according to Bicho et al. [105], with minor modifications. Five fruits per accession were selected and read directly on the surface using a colorimeter (Konica Minolta CR-400, Konica Minolta Inc., Tokyo, Japan) calibrated with a standard white tile (Y = 87.01; x = 0.3169; y = 0.3236), illuminant C, and a 2° observer. For parchment and green coffee, ten representative beans were placed in the measurement cell. The color coordinates L* (lightness), a* (green–red), b* (blue–yellow), C* (chroma), and *h°* (hue angle) were recorded using OnColor v5 software.

#### 5.5.3. Moisture Content

The moisture content of green coffee beans was determined according to NTP-ISO 6673 [106], with minor modifications. Five grams of the ground sample were weighed and subjected to oven drying at 105 °C for approximately 24 h or until constant weight was achieved. The analyses were performed in triplicate.

### 5.6. Extraction and Spectrophotometric Determination of Bioactive Compounds

Aqueous extracts were prepared following AOAC 2017.13 [107], with slight modifications. Ground coffee (50 mg) was dissolved in 50 mL of distilled water, ultrasonicated (Branson 3510, Branson Ultrasonics, Danbury, CT, USA) at room temperature for 10 min, and centrifuged (Hettich Mikro 22 R, Andreas Hettich GmbH & Co. KG, Tuttlingen, Germany) at 13,000 rpm for 10 min. From the supernatant, total phenolics, total flavonoids, and antioxidant activity (ABTS, DPPH, FRAP) were determined. Extractions and measurements were performed in triplicate.

#### 5.6.1. Total Phenolic Content

Total phenolics were determined according to AOAC 2017.13 [107], with minor modifications. An aliquot of 0.25 mL extract was mixed with 3.75 mL distilled water and 0.25 mL 2 N Folin–Ciocalteu reagent (Merck, Darmstadt, Germany). After 6 min, 0.75 mL of 20% (*w*/*v*) sodium carbonate was added, and the mixture was incubated at 30 ± 2 °C for 120 min. Absorbance was read at 765 nm using a UV–Vis spectrophotometer (Thermo Scientific Genesys 150, Thermo Fisher Scientific, Waltham, MA, USA). Phenolic content was calculated from a gallic acid calibration curve (25–150 mg L^−1^).

#### 5.6.2. Total Flavonoid Content

Total flavonoids were quantified following Abdeltaif et al. [108] and Haile et al. [109], with minor modifications. An aliquot of 0.5 mL extract was combined with 2.0 mL distilled water and 0.15 mL of 5% (*w*/*v*) sodium nitrite. After 5 min, 0.15 mL of 10% (*w*/*v*) aluminum chloride was added and incubated for 6 min, followed by 1.0 mL of 1 M sodium hydroxide and 1.2 mL distilled water. Absorbance was read at 510 nm, and total flavonoids were estimated from a catechin standard curve (20–100 mg L^−1^).

#### 5.6.3. Antioxidant Activity

ABTS assay was adapted from Re et al. [110] and Bressani et al. [111], with modifications. The ABTS radical solution was prepared by mixing 7 mM ABTS with 2.45 mM potassium persulfate and allowing the mixture to stand in the dark at room temperature for 16 h. One milliliter of this solution was diluted to 50 mL with ethanol and adjusted to an absorbance of 0.70 ± 0.02 at 750 nm. For measurements, 0.1 mL extract was mixed with 3 mL ABTS solution, incubated 7 min at room temperature, and read at 734 nm. Trolox (30–150 mg L^−1^) served as the reference standard.

DPPH was adapted from Brand-Williams et al. [112] and Bressani et al. [111], with minor modifications. A methanolic DPPH solution (100 µM) was adjusted to an absorbance of 0.70 ± 0.02 at 515 nm. Then, 100 µL extract was mixed with 2.9 mL DPPH solution and incubated in the dark at room temperature for 30 min; absorbance was read at 515 nm. A Trolox calibration curve (5–60 mg L^−1^) was used.

FRAP essay was adapted from Benzie et al. [113], with slight modifications. An aliquot of 0.1 mL extract was mixed with 3 mL FRAP reagent (300 mM acetate buffer, 10 mM TPTZ in 40 mM HCl, and 20 mM FeCl_3_ in a 10:1:1 ratio). After 4 min in the dark, absorbance was read at 593 nm. Ferrous sulfate standards (20–150 mg L^−1^) were used for quantification.

### 5.7. HPLC Analysis of Trigonelline, Chlorogenic Acids, and Caffeine

Aqueous extracts for chromatography were prepared following Sualeh et al. [7], with minor modifications. Ground coffee (50 mg) was mixed with 40 mL of ultrapure water preheated to 95 °C, and the mixture was shaken at 150 rpm for 20 min. Subsequently, the volume was adjusted to 50 mL with ultrapure water. The resulting solutions were filtered using Whatman No. 4 filter paper (Cytiva, Marlborough, MA, USA). Finally, a 1.5 mL aliquot of the aqueous extract was filtered through a nylon syringe filter (0.45 μm) directly into HPLC vials. All extractions and determinations were performed in triplicate.

Chromatographic separation followed Cho et al. [114], with modifications, on an HPLC system (Waters e2695, Waters Corporation, Milford, MA, USA) equipped with a PDA detector and a Hypersil Gold C18 column (150 × 4.6 mm, 5 µm). Injection volume was 20 µL; detection wavelength 280 nm; flow rate 1.5 mL min^−1^; column oven 35 °C. Elution used a gradient of solvent A (water/acetic acid, 98:2) and solvent B (water/acetonitrile/acetic acid, 68:30:2) as follows: 0–4 min, 4% B; 4–10 min, 5% B; 10–14 min, 95% B; 14–14.10 min, 0% B; 14.10–17 min, 0% B; 17–19 min, 4% B. Retention times (Supplementary Figure S5) were 1.6 min for trigonelline, 10.2 min for chlorogenic acids, and 12.8 min for caffeine. Quantification used external calibration curves (5–100 mg L^−1^) following Palmieri et al. [115], with minor modifications. Results are expressed as mg g^−1^ dry sample.

### 5.8. Multi-Trait Functional Selection Index

We developed a comprehensive, breeding-oriented composite index integrating ten variables (fruit yield per plant; green-bean weight, length, and width; total phenolics; total flavonoids; ABTS antioxidant activity; trigonelline; chlorogenic acids; caffeine). Accession means were ranked and binned into quintiles; traits were scored 1–5 (5 = best). The composite score (range 10–50) was obtained by summation. Ties at quintile boundaries were handled to preserve equal bin sizes. Full ranges are provided in Supplementary Table S1; standout genotypes are reported in the Results.

### 5.9. Statistical Analysis

All descriptive and inferential statistics reported in Table 1 were generated in GraphPad Prism v10.6 (GraphPad Software, San Diego, CA, USA). For each accession, laboratory replicates (triplicates for chemical/antioxidant assays; indicated subsamples for morphometry) were averaged, and accession-level means were used for analysis. Mean, SD, range, and CV were calculated; distributional assumptions were assessed with the Shapiro–Wilk test and homoscedasticity with Levene’s test. Group comparisons used one-way ANOVA followed by Tukey’s HSD (*p* < 0.05) when assumptions were met; otherwise, Kruskal–Wallis with Dunn–Bonferroni post hoc tests was applied. Pairwise associations were summarized as Spearman rank correlations (ρ), and correlation heatmaps/scatter-matrix plots were generated in RStudio 2025.09.0+387 using the *corrplot* and *MVN* packages. Interpretation emphasized effect sizes alongside *p*-values given the large sample size.

Multivariate analyses used accession-level means after autoscaling (mean-centering and unit-variance scaling). PCA was computed in RStudio 2025.09.0+387 via singular-value decomposition of the z-score matrix (*stats::prcomp*, center = TRUE, scale. = TRUE). Dimensionality was examined with scree plots of eigenvalues and parallel analysis to justify the number of components retained. Variable loadings, scores, and contribution metrics were cross-validated; biplots were generated with *readr, dplyr*, *ggplot2*, and *ggrepel*, adding 95% confidence ellipses by geographic origin where shown. Hierarchical clustering was performed in RStudio on the same autoscaled matrix using Euclidean distance (*stats::dist*) and Ward’s minimum-variance linkage (*stats::hclust*, method = “ward.D2”). The optimal number of clusters was selected by average silhouette width (*cluster::silhouette*) and summarized with *factoextra::fviz_nbclust* (method = “silhouette”); cluster membership was obtained with *stats::cutree*. Dendrograms were plotted with *dendextend*, *colorspace*, and *circlize* (circular layout for the manuscript). Cluster sizes/centroids and regional composition were tabulated from the resulting assignments.

Reproducibility controls included daily instrument calibration; reagent blanks for spectrophotometry; external standard curves prepared in matrix-matched solvents with verified linearity (R^2^ ≥ 0.99 where applicable); and white-tile/baseline checks for colorimetry prior to each batch. Missing values were rare (<5%); listwise deletion was used for PCA/HCA and pairwise deletion for Spearman matrices. PCA loading stability was confirmed via leave-one-out inspection (no changes to interpretation). Multivariate bubble plots were produced in GraphPad Prism v10.6 (GraphPad Software, San Diego, CA, USA).

## Figures and Tables

**Table 1:** Descriptive statistics for 40 agro-morphological, physicochemical, colorimetric, bioactive, and antioxidant activity traits in 150 coffee accessions from the INIA National Coffee Germplasm Collection (Peru).

Trait	Abbreviation	Unit	Mean	SD	CV	Min	Max	Median	Skewness	Kurtosis	Shapiro
Yield per plant	YPP	kg plant^−1^	0.90	0.55	61.58	0.14	2.64	0.79	0.99	0.83	<0.0001
Cherry fruit length	CFL	mm	15.53	1.00	6.46	12.55	20.18	15.42	0.68	2.49	0.0018
Cherry fruit width	CFW	mm	13.66	0.82	6.00	11.45	15.69	13.69	0.09	−0.34	0.6558
Cherry fruit thickness	CFT	mm	11.83	0.73	6.18	10.13	14.18	11.78	0.37	0.14	0.2373
Parchment coffee weight	PCW	g	0.19	0.03	16.66	0.14	0.37	0.19	2.25	8.85	<0.0001
Parchment coffee length	PCL	mm	11.98	1.02	8.52	8.77	16.17	11.92	1.35	4.71	<0.0001
Parchment coffee width	PCWD	mm	7.89	0.38	4.77	6.76	9.38	7.85	0.75	2.77	<0.0001
Parchment coffee thickness	PCT	mm	4.78	0.27	5.70	4.24	5.83	4.74	1.13	2.48	<0.0001
Green coffee weight	GCW	g	0.16	0.03	16.24	0.10	0.28	0.16	1.73	6.35	<0.0001
Green coffee length	GCL	mm	9.63	0.77	7.97	7.93	13.00	9.58	1.68	5.91	<0.0001
Green coffee width	GCWD	mm	6.72	0.33	4.95	5.92	8.20	6.72	0.78	2.37	0.0006
Green coffee thickness	GCT	mm	3.68	0.20	5.42	3.12	4.20	3.67	0.17	0.06	0.6541
Cherry coffee color L*	CCCL	Coordinate	37.50	7.49	19.97	30.54	60.05	35.05	1.95	2.68	<0.0001
Cherry coffee color a*	CCCA	Coordinate	22.16	7.48	33.74	−0.97	30.81	24.15	−2.07	3.20	<0.0001
Cherry coffee color b*	CCCB	Coordinate	16.82	8.98	53.39	8.38	44.70	13.92	2.07	3.13	<0.0001
Cherry coffee color C*	CCCC	Coordinate	29.92	4.87	16.26	20.61	45.00	28.82	1.17	1.32	<0.0001
Cherry coffee color *h°*	CCCH	Angle	35.29	19.04	53.94	21.17	91.61	28.51	2.21	3.41	<0.0001
Parchment coffee color L*	PCCL	Coordinate	51.26	1.71	3.34	46.23	55.61	51.23	0.02	0.13	0.9517
Parchment coffee color a*	PCCA	Coordinate	2.38	0.87	36.50	0.55	4.56	2.22	0.55	−0.26	0.0004
Parchment coffee color b*	PCCB	Coordinate	17.81	1.47	8.26	13.09	21.78	17.76	−0.23	0.76	0.1355
Parchment coffee color C*	PCCC	Coordinate	17.99	1.55	8.61	13.14	22.17	17.90	−0.13	0.63	0.1800
Parchment coffee color *h°*	PCCH	Angle	82.56	2.29	2.78	77.06	87.94	82.72	−0.25	−0.42	0.1342
Green Coffee Color L*	GCCL	Coordinate	44.60	1.58	3.54	39.01	48.88	44.59	−0.19	0.96	0.0509
Green Coffee Color a*	GCCA	Coordinate	0.94	0.24	25.29	0.39	1.59	0.92	0.38	−0.06	0.0410
Green Coffee Color b*	GCCB	Coordinate	9.92	0.85	8.56	6.72	12.07	9.83	−0.33	0.78	0.1693
Green Coffee Color C*	GCCC	Coordinate	9.97	0.85	8.55	6.76	12.15	9.90	−0.33	0.79	0.1816
Green Coffee Color *h°*	GCCH	Angle	84.64	1.30	1.53	81.20	87.72	84.71	−0.16	−0.11	0.8360
Soluble solids content initial	IB	°Brix	19.31	1.96	10.14	15.23	24.50	19.30	0.27	−0.16	0.0788
Soluble solids content final	FB	°Brix	12.75	1.72	13.50	8.53	16.13	12.95	−0.40	−0.46	0.0066
Initial pH	IPH	Value	5.25	0.39	7.45	4.44	6.28	5.19	0.37	−0.61	0.0069
Final pH	FPH	Value	4.03	0.36	8.90	3.08	4.78	4.09	−0.41	−0.22	0.0381
Green coffee humidity	GCH	%	6.89	0.64	9.35	5.22	8.64	6.94	−0.11	−0.39	0.5635
Total phenolic content ^(^*^)^	TPC	mg GAE g^−1^	45.53	4.07	8.94	32.73	57.78	44.99	0.14	0.75	0.0702
Total flavonoid content ^(^*^)^	TFC	mg CE g^−1^	40.01	3.81	9.53	28.82	50.68	39.78	−0.23	0.42	0.5024
ABTS ^(^*^)^	ABTS	µmol TE g^−1^	206.00	21.22	10.30	146.50	259.60	205.70	−0.07	−0.20	0.3863
DPPH ^(^*^)^	DPPH	µmol TE g^−1^	223.70	41.22	18.43	114.70	321.10	232.10	−0.77	0.09	<0.0001
FRAP ^(^*^)^	FRAP	µmol Fe^+2^ g^−1^	383.80	73.55	19.17	156.30	520.40	391.80	−0.82	0.59	<0.0001
Chlorogenic acid ^(^*^)^	CGA	mg g^−1^	42.88	5.51	12.85	30.50	55.42	42.70	−0.03	−0.55	0.4237
Trigonellin ^(^*^)^	TGN	mg g^−1^	12.08	1.51	12.52	9.04	15.74	11.85	0.05	−0.80	0.0171
Caffeine ^(^*^)^	CAF	mg g^−1^	11.84	1.18	9.96	8.57	15.39	11.76	0.13	0.36	0.5022

^(^*^)^ Results expressed on a dry weight basis.

**Table 2:** Cluster membership, regional representation, and defining trait profiles from Ward’s hierarchical clustering (Euclidean distance) of 150 coffee accessions.

Cluster	n	Provenance Zones	Accessions List (PER Code)	Associated Traits
Cluster 1	34	Cajamarca	PER1002171, PER1002172, PER1002174, PER1002176, PER1002177, PER1002178, PER1002180, PER1002181, PER1002182, PER1002183, PER1002184, PER1002186, PER1002187, PER1002190, PER1002193, PER1002194, PER1002195, PER1002196, PER1002197, PER1002199, PER1002200, PER1002202, PER1002203, PER1002204, PER1002206, PER1002207, PER1002208, PER1002211, PER1002212, PER1002216, PER1002217, PER1002219, PER1002220	Bioactive compounds and antioxidant activity
		Amazonas	PER1002231	
Cluster 2	5	Cajamarca	PER1002179	Agro-morphologic
		Pasco	PER1002298, PER1002305, PER1002310	
		Ucayali	PER1002318	
Cluster 3	94	Cajamarca	PER1002198, PER1002205, PER1002213, PER1002222	Yield, fermentation and bioactive compounds
		Amazonas	PER1002225, PER1002226, PER1002227, PER1002228, PER1002229, PER1002230, PER1002232, PER1002233, PER1002234, PER1002235, PER1002236, PER1002237, PER1002238, PER1002239, PER1002240, PER1002243, PER1002244, PER1002245, PER1002246, PER1002247, PER1002248, PER1002249, PER1002250, PER1002252, PER1002254	
		Junín	PER1002257, PER1002258, PER1002259, PER1002261, PER1002263, PER1002264, PER1002265, PER1002267, PER1002268, PER1002269, PER1002270, PER1002271, PER1002272, PER1002273, PER1002274, PER1002275, PER1002276, PER1002277, PER1002278, PER1002280, PER1002281, PER1002282, PER1002283, PER1002285, PER1002287, PER1002313	
		Pasco	PER1002288, PER1002289, PER1002290, PER1002291, PER1002292, PER1002293, PER1002294, PER1002295, PER1002297, PER1002299, PER1002300, PER1002301 PER1002302, PER1002303, PER1002304, PER1002306, PER1002307, PER1002308, PER1002309, PER1002311, PER1002312	
		Huánuco	PER1002314, PER1002315, PER1002316, PER1002317, PER1002320, PER1002321, PER1002322, PER1002323, PER1002324, PER1002325, PER1002327, PER1002328, PER1002329, PER1002331, PER1002332, PER1002336, PER1002339	
		Ucayali	PER1002319	
Cluster 4	17	Cajamarca	PER1002214, PER1002215, PER1002223	Cherry color
		Amazonas	PER1002241, PER1002251, PER1002253	
		Junín	PER1002255, PER1002256, PER1002262, PER1002266, PER1002279	
		Pasco	PER1002296	
		Huánuco	PER1002326, PER1002330, PER1002333, PER1002335, PER1002337	

## Data Availability

The original contributions presented in this study are included in the article/Supplementary Material. Further inquiries can be directed to the corresponding author.

## Supplementary Materials

The following supporting information can be downloaded at: https://www.mdpi.com/article/10.3390/plants15010013/s1. Figure S1: Scree plot of bivariate correlations between agro-morphological and phytochemical parameters; Figure S2: Proportion of variance, individual and cumulative, of principal component analysis of phytochemical and agro-morphological parameters; Figure S3: Proportion of variance individual and cumulative of principal component analysis of phytochemical, color and fermentation parameters; Figure S4: Determination of optimal cluster number based on average silhouette width for multivariate trait data; Figure S5: Chromatogram of trigonelline, chlorogenic acid, and caffeine in standards (A) and green coffee sample (B); Table S1: Multi-trait functional breeding selection index for 150 Coffea arabica accessions. Table S2. Provenance data and mean values of 40 agro-morphological, physicochemical, colorimetric, bioactive, and antioxidant activity traits in 150 Coffea arabica L. accessions from the INIA National Coffee Germplasm Collection (Peru).
