# Supplementary material for: Morpho-Physicochemical, Bioactive, and Antioxidant Profiling of Peruvian Coffea arabica L. Germplasm Reveals Promising Accessions for Agronomic and Nutraceutical Breeding
Source: Plants (Basel). 2025 Dec 19;15(1):13. doi: 10.3390/plants15010013 (PMC12787891; doi:10.3390/plants15010013)
Supplement: Supplementary file 1 [file plants-15-00013-s001.zip › plants-3968756-supplementary.pdf]

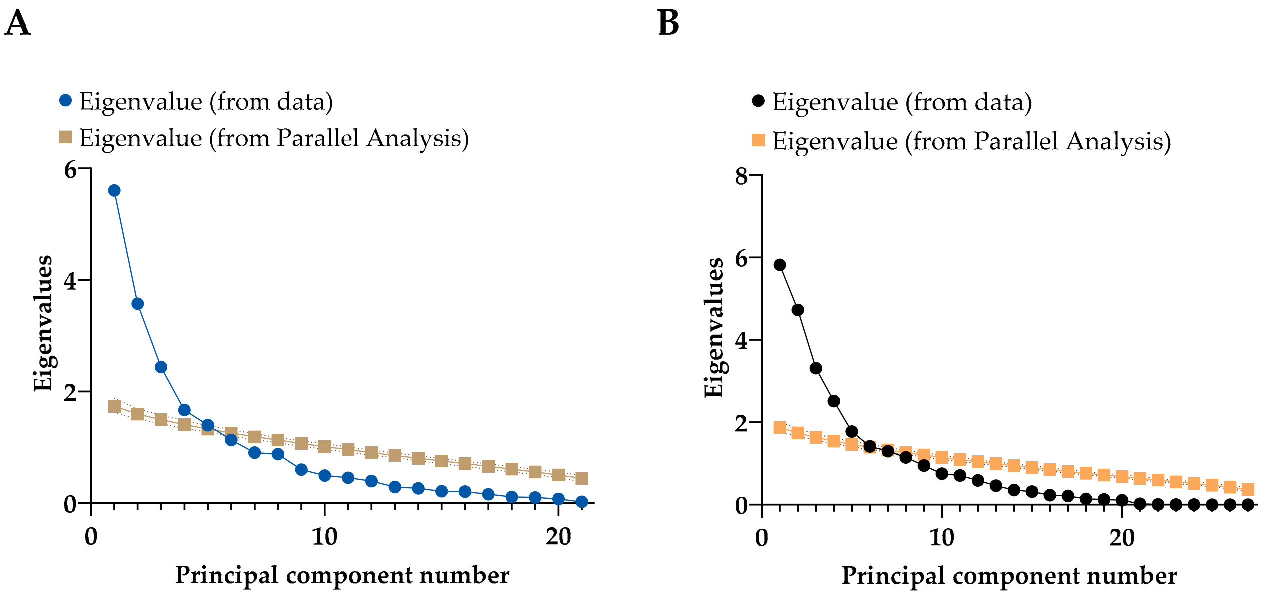

Figure S1. Scree plot of bivariate correlations between agro-morphological and phytochemical parameters (A) and color and fermentation with phytochemical parameters (B).

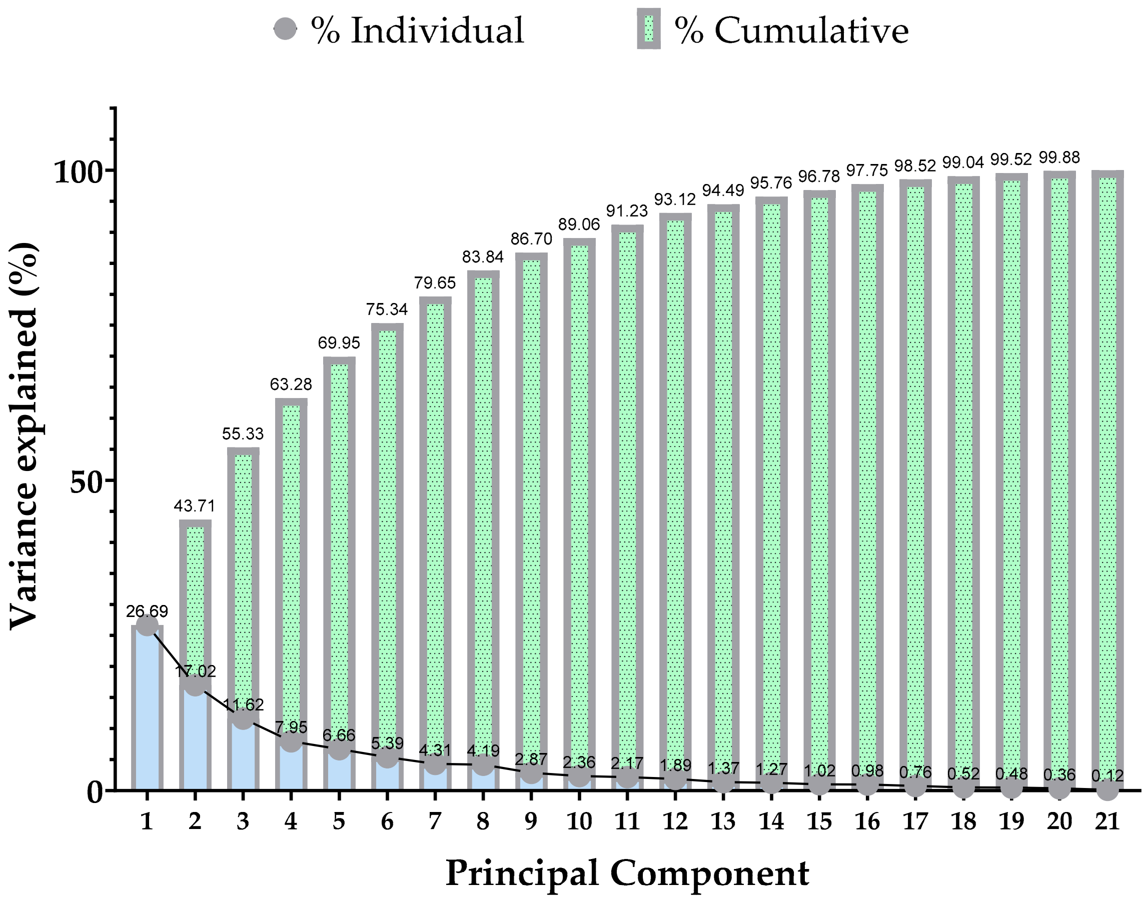

Figure S2. Proportion of variance, individual and cumulative, of principal component analysis of phytochemical and agromorphological parameters.

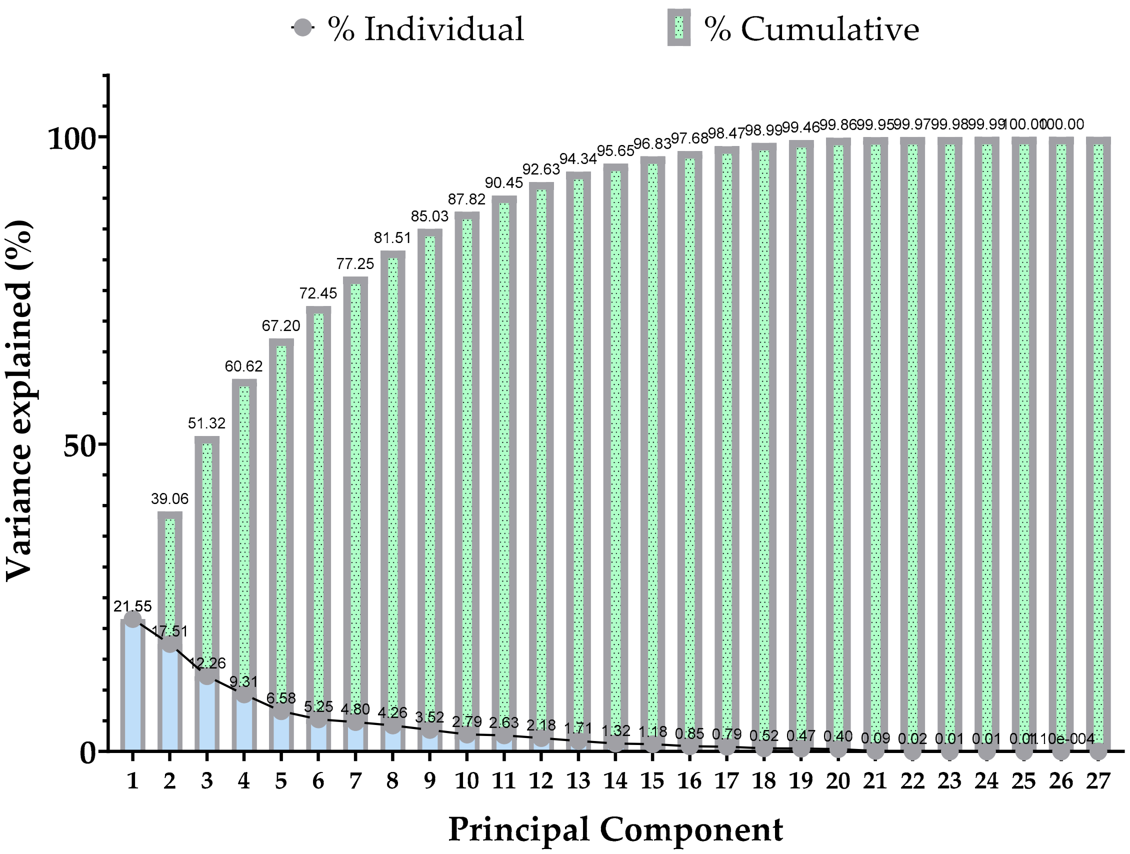

Figure S3. Proportion of variance individual and cumulative of principal component analysis of phytochemical, color and fermentation parameters.

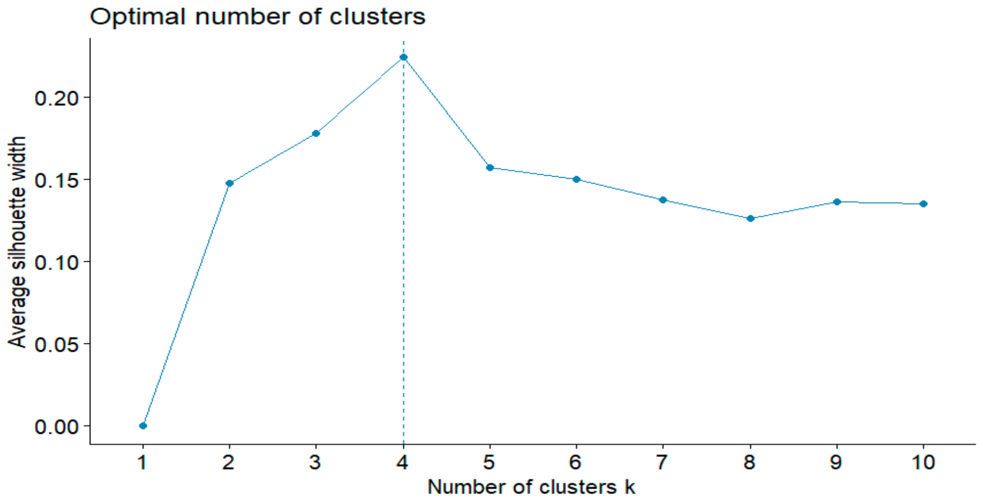

Figure S4. Determination of optimal cluster number based on average silhouette width for multivariate trait data.

Table S1. Multi-trait functional breeding selection index for 150 *Coffea arabica* accessions.

Accessions were ranked per trait across the full panel and assigned quintile scores (1 = lowest quintile; 5 = highest). The composite "SUM SCORE" equals the sum of ten per-trait scores (range 10–50). Abbreviations: ACC, accession ID; CGA, chlorogenic acid; CAF, caffeine; TGN, trigonelline; TPC, total phenolic content; TFC, total flavonoid content; ABTS, antioxidant capacity by the ABTS assay; YPP, yield per plant; GCW, green coffee bean weight; GCL, green coffee bean length; GCWD, green coffee bean width; SUM SCORE, composite index (10–50).

ACC	CGA	CAF	TGN	TPC	TFC	ABTS	YPP	GCW	GCL	GCWD	SUM SCORE
PER1002197	4	5	5	5	4	5	5	5	5	5	48
PER1002222	4	5	4	5	5	5	4	5	4	5	46
PER1002207	4	5	3	4	5	4	5	5	5	5	45
PER1002288	5	1	3	5	5	5	3	5	5	5	42
PER1002292	5	4	4	5	5	5	3	4	4	3	42
PER1002184	4	1	4	5	5	5	4	4	4	5	41
PER1002313	5	2	5	4	4	5	5	4	3	4	41
PER1002290	5	3	5	5	5	5	2	4	5	2	41
PER1002320	5	4	5	4	4	4	2	5	3	5	41
PER1002237	3	4	2	3	5	5	5	5	5	4	41
PER1002240	5	5	3	4	5	4	2	5	3	5	41
PER1002172	5	5	4	5	2	3	4	4	5	4	41
PER1002323	5	4	4	4	2	5	3	5	4	4	40
PER1002314	3	4	5	3	3	4	4	5	5	4	40
PER1002195	2	4	3	4	5	5	2	5	5	5	40
PER1002294	4	5	4	3	4	2	3	5	5	5	40
PER1002274	4	5	5	3	4	5	2	4	5	3	40
PER1002226	3	5	2	5	5	5	5	4	2	4	40
PER1002259	3	5	1	5	5	5	4	5	4	3	40
PER1002174	4	4	4	5	2	3	5	4	4	4	39
PER1002202	4	5	2	4	4	5	4	4	3	4	39
PER1002216	4	3	5	4	4	4	5	3	2	4	38
PER1002254	1	4	4	4	4	5	3	5	4	4	38
PER1002276	5	5	4	5	5	4	1	3	4	2	38
PER1002234	4	1	3	5	5	5	2	4	4	4	37
PER1002287	5	2	5	4	4	5	5	3	2	2	37
PER1002186	4	2	2	3	3	4	5	5	3	5	36
PER1002187	4	4	4	3	4	4	2	3	4	4	36
PER1002235	4	4	2	5	5	5	1	4	1	5	36
PER1002311	5	5	5	2	3	2	2	4	5	3	36
PER1002275	5	5	4	4	5	3	1	3	4	2	36
PER1002289	5	1	5	5	5	4	1	3	3	3	35
PER1002205	5	2	4	4	5	5	1	3	4	2	35
PER1002225	2	2	3	5	5	5	2	3	3	5	35

ACC	CGA	CAF	TGN	TPC	TFC	ABTS	YPP	GCW	GCL	GCWD	SUM SCORE
PER1002321	4	3	5	5	2	2	4	3	3	4	35
PER1002336	4	3	3	4	4	3	2	4	5	3	35
PER1002262	2	3	1	5	4	5	1	5	5	4	35
PER1002236	3	4	1	5	5	5	2	3	4	3	35
PER1002232	1	5	4	4	4	4	1	4	3	5	35
PER1002179	2	2	4	3	4	3	1	5	5	5	34
PER1002239	1	4	1	4	5	5	4	4	2	4	34
PER1002190	5	5	5	4	3	5	4	1	1	1	34
PER1002305	4	5	4	1	2	2	1	5	5	5	34
PER1002229	2	5	2	5	5	5	2	3	2	3	34
PER1002200	3	1	4	3	3	4	5	3	3	4	33
PER1002243	3	1	1	3	4	4	3	5	4	5	33
PER1002219	1	2	3	3	3	3	5	4	5	4	33
PER1002206	3	3	1	3	3	3	4	5	3	5	33
PER1002227	3	5	3	5	5	5	2	2	1	2	33
PER1002295	4	1	3	3	3	2	1	5	5	5	32
PER1002322	5	2	4	4	3	4	3	2	2	3	32
PER1002182	2	3	4	3	3	3	5	3	3	3	32
PER1002253	5	4	2	5	5	5	2	1	2	1	32
PER1002312	5	4	5	4	1	2	5	2	2	2	32
PER1002171	1	4	5	3	2	1	2	4	5	5	32
PER1002301	5	5	5	1	1	2	5	3	2	3	32
PER1002230	2	5	2	5	1	2	3	5	3	4	32
PER1002298	2	5	5	1	2	1	1	5	5	5	32
PER1002271	5	1	4	3	4	5	4	2	1	2	31
PER1002299	5	1	4	4	5	4	4	1	1	2	31
PER1002306	3	1	2	2	3	4	3	4	4	5	31
PER1002193	4	3	5	4	5	5	1	1	1	2	31
PER1002263	1	3	3	1	1	3	4	5	5	5	31
PER1002283	5	4	3	3	4	2	2	3	3	2	31
PER1002291	3	4	3	1	2	1	3	5	5	4	31
PER1002318	4	5	1	1	1	1	3	5	5	5	31
PER1002198	4	5	4	4	3	4	4	1	1	1	31
PER1002310	2	5	5	1	1	1	3	5	5	3	31
PER1002272	5	1	5	1	1	1	3	4	5	4	30
PER1002223	3	3	2	4	4	4	5	2	2	1	30
PER1002255	1	3	3	5	1	1	5	4	4	3	30
PER1002303	5	4	4	1	1	2	4	3	4	2	30
PER1002203	2	4	1	2	3	3	2	4	5	4	30
PER1002325	5	5	3	2	2	2	3	2	3	3	30
PER1002196	4	5	3	5	3	4	3	1	1	1	30

ACC	CGA	CAF	TGN	TPC	TFC	ABTS	YPP	GCW	GCL	GCWD	SUM SCORE
PER1002217	1	5	4	4	5	5	2	2	1	1	30
PER1002279	3	1	3	3	3	4	5	2	1	4	29
PER1002270	3	1	5	2	2	3	5	3	3	2	29
PER1002267	1	1	5	3	3	4	2	4	3	3	29
PER1002327	2	2	2	2	2	3	4	5	3	4	29
PER1002250	1	3	2	5	5	4	2	2	2	3	29
PER1002282	4	4	2	2	3	1	1	4	3	5	29
PER1002246	2	4	1	2	2	3	5	3	4	3	29
PER1002183	4	1	5	3	3	3	3	2	2	2	28
PER1002278	3	2	5	4	1	1	3	3	4	2	28
PER1002277	2	2	5	1	1	1	2	5	5	4	28
PER1002266	1	2	3	2	3	4	2	4	3	4	28
PER1002300	5	3	5	1	1	2	3	2	2	4	28
PER1002329	3	3	3	2	4	2	4	2	4	1	28
PER1002208	2	3	2	4	4	4	5	1	1	2	28
PER1002245	2	3	1	4	5	4	2	2	3	2	28
PER1002248	1	3	1	1	2	2	5	5	5	3	28
PER1002302	5	4	5	2	3	3	3	1	1	1	28
PER1002317	4	4	5	2	3	2	3	2	1	2	28
PER1002181	2	5	4	1	2	2	4	2	3	3	28
PER1002212	1	5	2	1	2	1	4	3	4	5	28
PER1002211	3	2	2	4	4	4	5	1	1	1	27
PER1002273	2	2	4	3	4	3	5	1	2	1	27
PER1002331	2	2	2	2	2	3	1	4	4	5	27
PER1002252	2	3	2	2	3	3	3	3	5	1	27
PER1002285	4	4	5	2	2	2	1	2	4	1	27
PER1002319	4	4	3	1	2	2	3	3	4	1	27
PER1002220	2	1	3	4	5	4	4	1	1	1	26
PER1002238	2	3	1	5	1	1	5	2	3	3	26
PER1002296	1	3	4	5	4	2	2	2	2	1	26
PER1002180	1	4	3	1	1	1	4	4	3	4	26
PER1002268	5	1	3	4	5	1	3	1	1	1	25
PER1002297	3	1	2	5	2	2	4	2	2	2	25
PER1002177	1	1	3	1	1	1	5	3	4	5	25
PER1002309	3	2	3	1	1	1	4	3	2	5	25
PER1002269	1	2	5	2	2	1	4	2	4	2	25
PER1002247	1	2	1	3	4	3	4	2	2	3	25
PER1002315	3	3	3	2	1	3	3	2	2	3	25
PER1002233	1	3	1	2	3	4	3	3	2	3	25
PER1002176	3	1	2	5	3	2	5	1	1	1	24
PER1002293	3	2	4	2	2	1	1	2	2	5	24

ACC	CGA	CAF	TGN	TPC	TFC	ABTS	YPP	GCW	GCL	GCWD	SUM SCORE
PER1002213	2	2	1	3	4	4	2	2	3	1	24
PER1002257	1	2	1	1	1	1	2	5	5	5	24
PER1002194	2	3	2	1	1	3	4	3	2	3	24
PER1002241	1	3	1	2	2	3	4	3	2	3	24
PER1002214	1	5	2	2	2	3	4	1	3	1	24
PER1002256	2	2	2	4	4	3	3	1	1	1	23
PER1002228	2	2	2	3	3	4	1	2	3	1	23
PER1002264	1	2	2	2	2	1	1	4	5	3	23
PER1002265	1	3	4	1	1	1	1	3	4	4	23
PER1002326	4	4	4	2	2	2	1	1	2	1	23
PER1002328	2	4	1	1	1	1	5	3	3	2	23
PER1002316	4	1	1	3	2	4	1	2	2	2	22
PER1002324	3	1	1	2	1	2	2	4	3	3	22
PER1002178	3	1	1	3	4	3	4	1	1	1	22
PER1002308	3	2	4	2	1	2	4	1	1	2	22
PER1002215	3	3	3	3	1	1	5	1	1	1	22
PER1002204	2	3	2	1	1	3	2	2	2	4	22
PER1002199	2	3	3	1	1	2	5	1	2	2	22
PER1002249	1	3	1	2	2	2	5	2	2	2	22
PER1002304	3	4	5	1	1	1	3	1	1	2	22
PER1002337	3	4	1	3	4	2	1	1	2	1	22
PER1002281	5	1	1	5	1	1	3	1	1	2	21
PER1002261	1	1	1	2	3	1	1	4	4	3	21
PER1002333	4	1	1	3	4	3	1	1	1	1	20
PER1002335	2	2	1	1	1	1	1	4	4	3	20
PER1002339	2	2	2	1	2	2	3	1	1	4	20
PER1002244	1	2	1	3	3	3	1	2	3	1	20
PER1002307	5	3	3	1	3	1	1	1	1	1	20
PER1002332	3	1	1	2	3	2	1	2	2	2	19
PER1002258	1	1	2	1	2	1	1	3	4	3	19
PER1002330	3	2	2	1	3	2	2	1	1	2	19
PER1002251	1	1	1	2	4	3	2	1	1	2	18
PER1002231	1	2	2	3	1	3	3	1	1	1	18
PER1002280	4	1	3	2	2	1	1	1	1	1	17

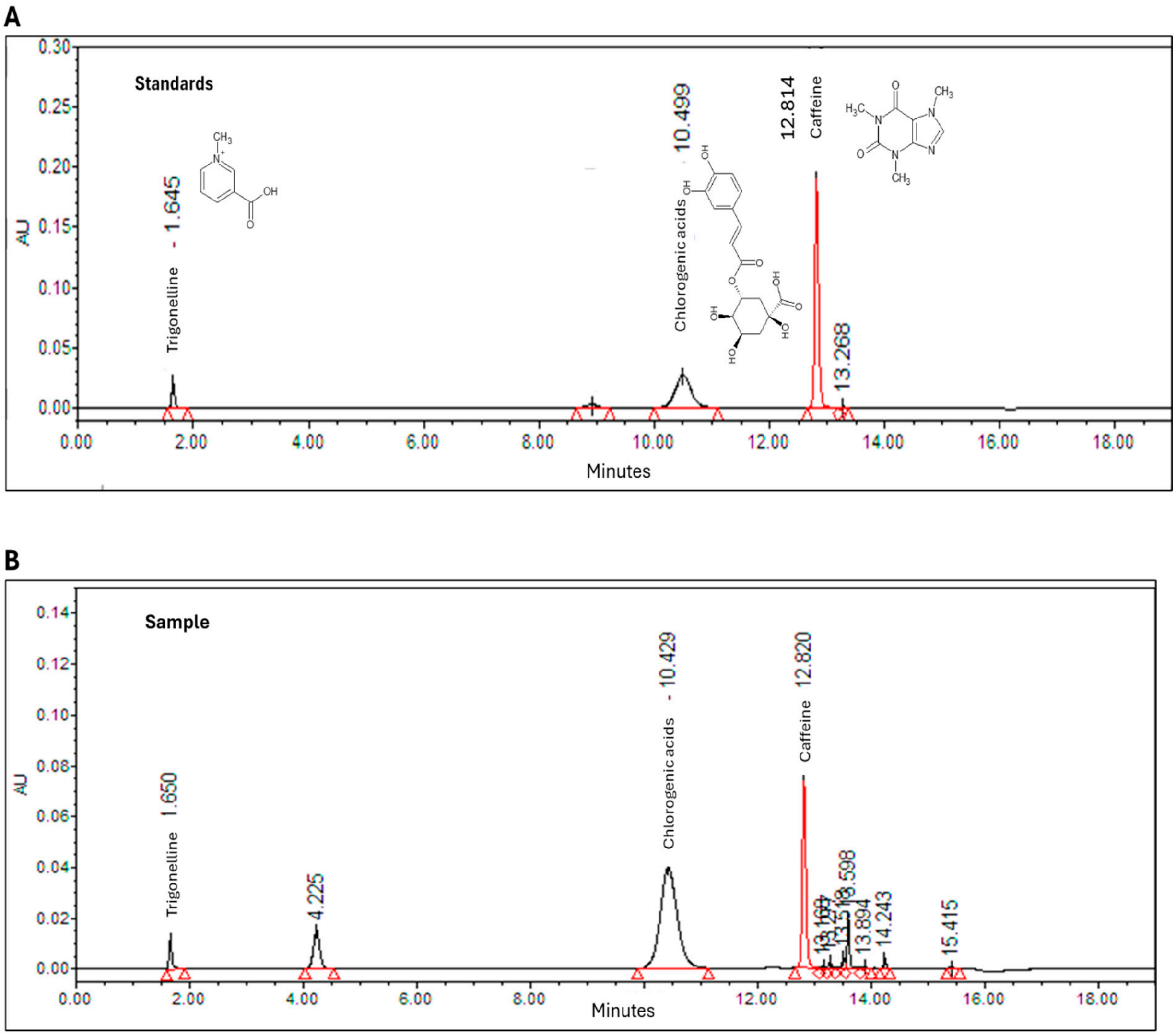

Figure S5. Chromatogram of trigonelline, chlorogenic acid, and caffeine in standards (A) and green coffee sample (B)
